# Modulating the gut microenvironment as a treatment strategy for irritable bowel syndrome: a narrative review

**DOI:** 10.1017/gmb.2022.6

**Published:** 2022-08-25

**Authors:** Cristina Iribarren, Lujain Maasfeh, Lena Öhman, Magnus Simrén

**Affiliations:** 1 Department of Microbiology and Immunology, Institute of Biomedicine, Sahlgrenska Academy, University of Gothenburg, Gothenburg, Sweden; 2 Department of Molecular and Clinical Medicine, Institute of Medicine, Sahlgrenska Academy, University of Gothenburg, Gothenburg, Sweden; 3 Center for Functional GI and Motility Disorders, University of North Carolina, Chapel Hill, NC, USA

**Keywords:** Antibiotics, dietary habits, faecal microbiota transplantation, irritable bowel syndrome, probiotics, prebiotics

## Abstract

Irritable bowel syndrome (IBS) is a disorder of gut–brain interaction with a complex pathophysiology. Growing evidence suggests that alterations of the gut microenvironment, including microbiota composition and function, may be involved in symptom generation. Therefore, attempts to modulate the gut microenvironment have provided promising results as an indirect approach for IBS management. Antibiotics, probiotics, prebiotics, food and faecal microbiota transplantation are the main strategies for alleviating IBS symptom severity by modulating gut microbiota composition and function (eg. metabolism), gut barrier integrity and immune activity, although with varying efficacy. In this narrative review, we aim to provide an overview of the current approaches targeting the gut microenvironment in order to indirectly manage IBS symptoms.

## Introduction

Irritable bowel syndrome (IBS), with a worldwide prevalence of 4 per cent (female-to-male ratio of 2:1), is an extensively researched disorder of gut–brain interaction (DGBI), formerly known as functional gastrointestinal (GI) disorders (Sperber et al., 2021). The diagnosis is based on the clinical history and symptoms. According to the Rome IV criteria, these patients are defined by the presence of chronic or recurrent abdominal pain associated with defecation and/or altered bowel habits, in the absence of positive findings on the limited number of tests recommended to exclude organic diseases. Based on bowel habits, patients are categorised into predominant constipation (IBS-C), predominant diarrhoea (IBS-D), mixed bowel habits (IBS-M) or IBS unclassified (IBS-U) (Lacy et al., 2016). Patients also often report concomitant symptoms and comorbidities such as bloating, abdominal distension, overlapping upper GI symptoms and extra-intestinal or psychological conditions (Enck et al., 2016; Lacy et al., 2016). IBS is not life-threatening but it greatly impacts the quality of life of the patients and via high health care consumption and reduced work productivity, also the society (Canavan et al., 2014).

An intricate pathophysiology with absence of organic disease or well-defined biological markers is characteristic of this disorder with unknown aetiology (Enck et al., 2016). Still, the gut-brain axis and its bidirectional interaction seem to be a cornerstone for symptom generation, hence the new term for these disorders, DGBI (Drossman, 2016). In addition, the generation of the hallmark IBS symptoms may be influenced by other factors, such as visceral hypersensitivity (Posserud et al., 2007), altered GI motility (Simrén et al., 2000), increased intestinal permeability (Piche et al., 2009) and low-grade mucosal inflammation (Spiller, 2004), along with alterations in the gut microenvironment, including gut microbiota (Öhman et al., 2015).

### Gut microbiota and IBS

The link between the gut microbiota and IBS is supported by multiple studies and clinical observations. A proportion of patients with IBS develops symptoms following a resolved bacterial infection (post-infection IBS) (Barbara et al., 2019), presumably linked to alterations of gut microbiota composition (Jalanka-Tuovinen et al., 2014). Further, the use of systemic antibiotics for non-GI conditions may also have a negative impact on gut microbiota and increase the risk of various DGBI (Paula et al., 2015). On the contrary, targeting gut microbiota using non-absorbable antibiotics or probiotics holds promise as a treatment strategy in IBS (Ford et al., 2018).

Even though there are inconsistent findings in the literature, a subset of IBS patients seems to display an altered gut microbiota composition compared with healthy individuals (Liu et al., 2017; Pittayanon et al., 2019), which can be associated with clinical and psychological parameters (Jeffery et al., 2012), IBS severity or low microbial richness (Tap et al., 2017). Interestingly, specific bacteria have also been associated with IBS (eg. decrease of *Bifidobacterium* and *Faecalibacterium* and increase of the genus *Bacteroides* as compared to healthy subjects), as reviewed in Pittayanon et al. (2019). Pathogenic bacteria, such as *Brachyspira*, could potentially also play a role in the pathogenesis (Jabbar et al., 2021). Despite multiple studies indicating involvement of altered microbiota in IBS pathogenesis, the challenge lies in attributing disease causality to a gut microbiota profile or specific bacterial taxa. It is worth noting that gut microbiota in IBS seems unstable over time compared to healthy subjects (Mättö et al., 2005) and under the influence of exogenous factors (eg. diet and antibiotics) (Bhattarai et al., 2017) as well as bowel habits that shift the intestinal microenvironment (Durbán et al., 2013), making it unclear whether altered microbiota is a cause, consequence, or both, of IBS. Altogether, these findings contribute to the understanding of IBS and potentially facilitate the characterisation of patients in regards to prognosis and response to treatments in the foreseeable future (Jeffery et al., 2012).

### The link between metabolites and IBS

Metabolites ensure the host-microbiota crosstalk and are involved in various biological functions in the gut, including providing energy for epithelial cells, maintenance of intestinal barrier function and nutrient absorption (Nicholson et al., 2012). Earlier studies have indicated that some IBS patients have altered levels of specific metabolite classes, such as short-chain fatty acids (SCFAs), bile acids and amino acids (Duboc et al., 2012; Tana et al., 2010; Zhang et al., 2019). More recent studies making use of untargeted metabolomics analyses have also revealed alterations in the metabolite profiles in serum (Xu et al., 2020), urine (Jeffery et al., 2020; Liu et al., 2020), and faeces (Ahluwalia et al., 2021; Jeffery et al., 2020; Lee et al., 2020; Ponnusamy et al., 2011; Zhu et al., 2019) of IBS patients. While there is no clear consensus on these changes being the cause or effect of IBS, the metabolome alterations have been found to be associated with gut microbiota composition (Jeffery et al., 2020; Lee et al., 2020; Xu et al., 2020; Zhu et al., 2019), IBS symptoms (Liu et al., 2020; Xu et al., 2020; Zhu et al., 2019) as well as psychological well-being (Liu et al., 2020). Although the metabolome profile alone fails to discriminate between IBS subtypes (Jeffery et al., 2020), combined with faecal microbiota pattern a separation between IBS-C and IBS-D can be observed (Ahluwalia et al., 2021). Metabolite alterations during IBS flares further support the link between microbial metabolism and IBS severity, as shown in a recent longitudinal study (Mars et al., 2020).

**Figure 1:**
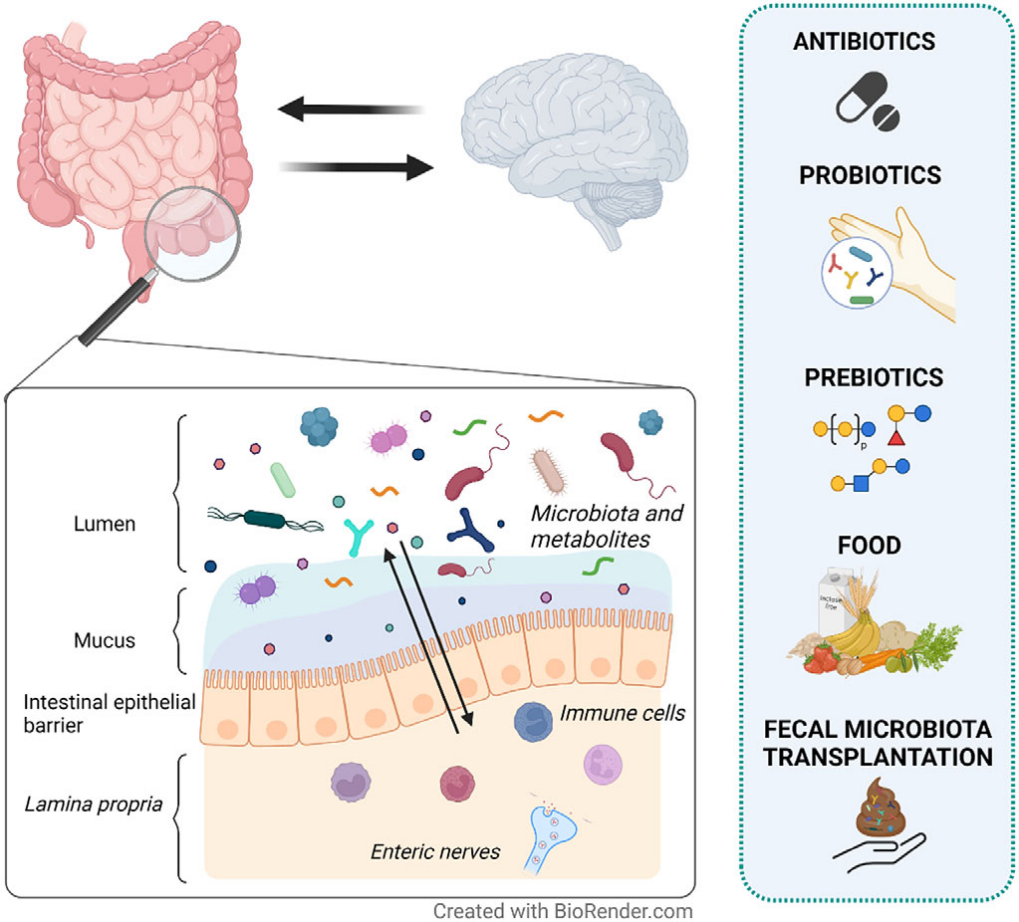
Therapeutic strategies proposed to modulate the gut microenvironment in patients with irritable bowel syndrome (IBS). IBS is a disorder of gut–brain interaction where alterations in either direction may influence the opposite end. The intestinal epithelial barrier separates the content of the lumen (gut microenvironment) from the underlying lamina propria. Local immune cells and enteric nerves located in the lamina propria independently or collaboratively sense and respond to signals in the gut microenvironment. Therefore, changes in the gut microenvironment are suggested to play a role in symptom generation and other factors involved in the pathophysiology of IBS. Although with mechanisms yet far from fully understood, antibiotics, probiotics, prebiotics, food and faecal microbiota transplantation (FMT) may influence the gut microenvironment (eg. microbiota and metabolites) and modulate symptoms in IBS patients.

In this narrative review, we aim to provide an overview of the current strategies targeting the gut microenvironment that are proposed as treatment options for DGBI (Figure 1). Particularly we focus on studies assessing the effects on gut microenvironment, including microbiota and metabolites, and its interaction with clinical symptoms in patients with IBS.

## Antibiotics

Antibiotics first showed benefits in patients with diagnosis of IBS and Small Intestinal Bacterial Overgrowth (SIBO), two entities with potentially overlapping symptoms (Pimentel et al., 2000). Generally, SIBO is diagnosed using non-invasive breath tests after intake of carbohydrates, most frequently lactulose, by quantifying excreted hydrogen and methane produced by microbial fermentation (Gasbarrini et al., 2009). This test has also been suggested to reflect alterations in microbiota composition in IBS patients (Pimentel et al., 2000). However, the link between IBS and SIBO is still controversial and part of the problem is related to the absence of a gold standard to define SIBO as well as poor performance of available tests (Aziz et al., 2017). However, small bowel microbial alterations seem to be present in patients with DGBI, but are not necessarily associated with SIBO, as it is currently defined. To date, the antibiotics neomycin, rifaximin and rifamycin have been the most investigated in IBS, with potential benefits regarding improvement in IBS symptoms (Ford et al., 2018).

### Neomycin

Little is known about the effects of the oral antibiotic neomycin on the gut microenvironment, but it has shown capacity to improve IBS symptoms and normalise the lactulose breath test (Pimentel et al., 2003). In patients with IBS-C presenting with excretion of methane, neomycin administration improved symptoms along with methane being eliminated on breath test (Pimentel et al., 2006), suggesting treatment-induced gut microbiota modulation.

### Rifaximin

The influence of the non-absorbable antibiotic rifaximin on the gut microenvironment has been more extensively explored. Administration of rifaximin can negatively affect microbial richness, but did not influence the faecal SCFAs or bile acid production in IBS patients without constipation with no evidence of SIBO (Acosta et al., 2016). When SIBO is concomitant, rifaximin has been shown to lower the levels of potentially pathogenic *Clostridium* spp., and increase *Faecalibaterium,* while inducing only modest changes in the overall faecal microbiota composition. However, these changes could not be associated with symptom amelioration or normalisation of the lactulose breath test due to the lack of a notable shift in the faecal microbiota composition and also probably the analysis of faecal rather than small intestinal microbiota (Soldi et al., 2015). Further, rifaximin administration in IBS-D patients seems to modify faecal microbiota composition and potentially also function, as well as eradicating SIBO (Zhuang et al., 2018). Potentially, these are the mechanisms of action underlying the relief of GI symptoms after rifaximin treatment (Zhuang et al., 2018). However, during recurrent symptoms and short-term repeated courses of rifaximin, its administration could have a short-term negative influence on certain faecal bacterial taxa (Fodor et al., 2019), although without apparent evidence of acquisition of antibiotic resistance in the long term (Pimentel et al., 2017). In parallel, numerous randomised clinical trials have reported promising results in treating IBS symptoms with rifaximin (Supplementary References: Rifaximin), although with some exceptions (Tuteja et al., 2019). Indeed, rifaximin seems to have a higher clinical response rate as primary treatment, as well as retreatment, than other antibiotics used for SIBO-IBS management. Besides, it can normalise breath test results (Soldi et al., 2015; Yang et al., 2008), especially in combination with neomycin in patients with abnormal levels of methane (Low et al., 2010; Pimentel et al., 2014), potentially reflecting changes in microbial fermentation (Sharara et al., 2006). Interestingly, gut microbiota composition (Li et al., 2020) and breath tests (Rezaie et al., 2019) have been proposed as promising prognostic tools for treatment response, although the link between microbiota and symptoms calls for further investigations.

### Rifamycin

Rifamycin SV is a poorly absorbed antibiotic that exerts its action in the distal small bowel and colon, at pH levels ≥7. To date, this antibiotic has not been associated with acquisition of multi-drug resistant bacteria (Steffen et al., 2018), presents *in vitro* proinflammatory properties (Rosette et al., 2019), and is approved in the US for treatment of traveller’s diarrhoea (Hoy, 2019). New formulations of rifamycin SV are currently under development for various GI diseases. These formulations are insignificantly absorbed in healthy individuals (Di Stefano et al., 2021) and demonstrate potential to improve abdominal pain and diarrhoea in IBS-D patients (Cosmo-Pharmaceuticals, 2021). More studies are still needed to decipher its effects on gut microenvironment, but, in the meantime, Rifamycin SV seems to be a promising new antibiotic therapy for the management of IBS.

### Current recommendations

Hitherto, rifaximin is the only antibiotic treatment used and approved for IBS-D, but its use is not universally accepted. In the US, rifaximin is approved for 2-week treatment of patients with IBS-D and recommended in the IBS management guidelines by the American College of Gastroenterology (Lacy et al., 2021). In contrast, rifaximin is currently not approved for use for IBS in Europe (Vasant et al., 2021). As stated above, rifamycin SV is only approved for traveller’s diarrhoea in the US, while studies to fully support its inclusion in IBS management and its impact on gut microenvironment are awaited.

**Figure 2:**
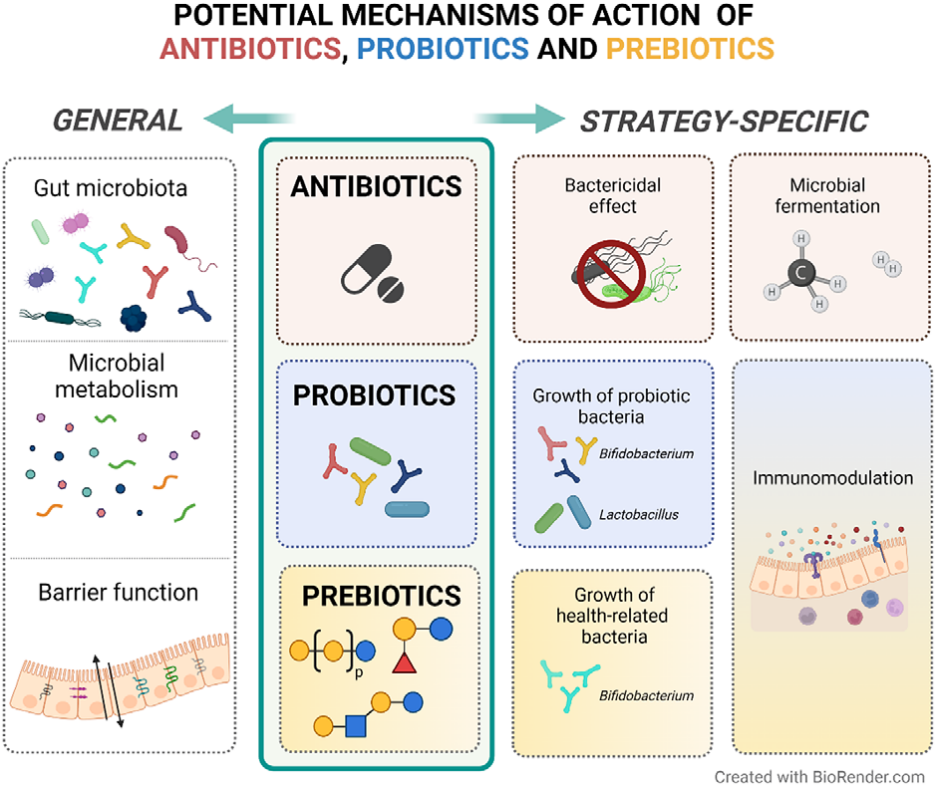
Potential mechanisms of action of antibiotics, probiotics and prebiotics in patients with IBS. In general, anti-, pro-, and pre-biotics are suggested to exert their therapeutic activity through favourable alterations on the gut microbiota composition, microbial metabolism products (eg. short-chain fatty acids) and gut barrier function. In particular, antibiotics can induce bacterial cell death and modulate methane and hydrogen production, which may reflect the fermentation activity of the microbiota. Both probiotics and prebiotics are suggested to have immunomodulatory effects on the host. Probiotics result in the growth of the administered bacteria, while prebiotics influence the growth of specific endogenous bacteria that are suggested to be related to health.

To summarise, non-absorbable antibiotics seem to be effective in the management of IBS symptoms. These antibiotics may exert their action through bactericidal effects by blocking essential biological pathways that lead to bacterial cell death (Floss and Yu, 2005; Jana and Deb, 2006). However, its use in IBS patients is still based on a hypothetical capacity of changing an imbalanced gut microbiota composition (Basseri et al., 2011) although other not yet well-defined mechanisms might be involved (Figure 2 – based on Floss and Yu (2005); Jana and Deb (2006); Pimentel (2016) – and Table 1). More studies focusing on the mechanisms of action of antibiotics and their effect on the gut microenvironment are needed to advance our understanding of how antibiotics may be used to manage IBS.

Box 1. Antibiotics – Key points

1. The use of non-absorbable antibiotics in IBS is not universally approved.
2. Clinical studies support the potential of certain non-absorbable antibiotics to improve IBS symptoms.
3. The mechanisms involved in symptom improvement may include bactericidal effect, changes in gut microbiota composition and microbial fermentation.

Box 2. Probiotics – Key points

1. Dependent on bacterial strains and doses, probiotics may have different mechanisms of action on gut microenvironment, including modulation of microbial composition and function, immune activity and barrier function.
2. The large diversity of study design and bacterial strains explain gaps of knowledge and weak support of probiotics as therapeutic options in IBS.
3. It is necessary to improve the characterisation of the mechanisms underlying the potential clinical benefits through studies using uniform study designs, approved clinical endpoints and valid mechanistic assessments.

**Table 1.** Overview of studies evaluating the effects of antibiotics on the gut microenvironment and clinical outcome in patients with IBS.

Antibiotic therapy	References	Design, study cohort (*n*), Rome criteria	Intervention	Main findings: gut microenvironment^a^	Main findings: symptoms^a^
Open-label antibiotics	Pimentel et al. (2000)	Retrospective study and open label, IBS patients with +LHBT and follow-up (*n* = 47), Rome I	10-day course of antibiotics based on the choice of the physician, followed by 7- to 10-day washout period	Reduction of hydrogen production after antibiotic treatment in 47 follow-up patients. Of them, 53% eradicated SIBO	SIBO eradication resulted in improvement of GI complaints in 48% of subjects, who no longer met Rome criteria for IBS
Neomycin	Pimentel et al. (2003)	RCT, IBS patients (*n* = 111), Rome I Healthy individuals (*n* = 15)	Neomycin (500 mg^b^) versus placebo for 10 days, followed by 7-day washout period	Abnormal LBT at baseline in 84% of IBS patients. Neomycin normalised breath test in 20% of patients with abnormal baseline LHBT. Methane excretion detected on LBT associated with severity of constipation (IBS-C)	Reduction of IBS symptoms (35% of patients in neomycin vs. 11.4% placebo) and bowel habit normalisation. Graded symptom reduction (61.7% neomycin and LBT normalisation vs. 34.4% neomycin and no LBT normalisation vs. 4.1% placebo)
	Pimentel et al. (2006)	RCT, IBS-C patients (*n* = 39), Rome I	Neomycin (500 mg) versus placebo for 10 days, followed by 7-day washout period	Ability of neomycin to eliminate methane on the breath test	General symptom improvement after neomycin treatment with greater efficacy in CH_4_-producers (67.6 vs. 32.7% H_2_-producers). Methane producers improved constipation and eliminated CH_4_ (44% neomycin vs. 5% placebo)
Rifaximin	Acosta et al. (2016)	RCT, IBS-nonC with no evidence of SIBO (*n* = 24), modified Rome III	Rifaximin (1.65 g) versus placebo for 14 days, followed by 5-day washout period	Modest effect on faecal microbiota, reduction of microbial richness but no changes in the proportion of faecal SCFA or bile acid proportion	No significant effects within the first 24 hours after rifaximin intervention. Acceleration of colonic transit after 48 hours
	Pimentel et al. (2017)	Open label + RCT, IBS-D patients (*n* = 103), Rome III	Open label for 2 weeks, followed by 4-week washout period. If symptom recurrence (*n* = 73), two 2-week course with Rifaximin (1.65 g) versus placebo, with 10 weeks between cycles	No apparent effect on antibiotic resistance to other non-absorbable antibiotics in the long-term	Not investigated
	Fodor et al. (2019)	Open label + RCT, IBS-D patients (*n* = 103), Rome III	Open label for 2 weeks, followed by 4-week washout period. If symptom recurrence, two 2-week course with Rifaximin (1.65 g) versus placebo, with 10 weeks between cycles	Modest and temporary decrease in the relative abundance of up to seven bacterial taxa associated with the treatment	Not investigated
	Tuteja et al. (2019)	RCT, Gulf war veterans with IBS-nonC (*n* = 50), Rome III	Rifaximin (1.1 g) versus placebo	Rifaximin treatment was not associated with normalisation of LHBT (7% rifaximin vs. 22% placebo, *p* = 0.54)	No improvement of IBS symptoms, or QoL in Gulf war veterans with IBS
	Yang et al. (2008)	Retrospective chart review, IBS patients and +LBT, and at least one follow-up visit (*n* = 98), Rome I	At least one course of Rifaximin (1.2 g or other doses) for 10 days versus other antibiotics	Normalisation of LBT predicted response to rifaximin (81%)	Greater improvement of IBS symptoms of rifaximin versus other antibiotics in patients with abnormal LBT. Large clinical response of rifaximin as first choice (69% vs. 38% neomycin) and higher success in cases of recurrence (75%)
	Low et al. (2010)	Retrospective chart review, IBS patients and CH_4_+ (*n* = 69)	Rifaximin (1.2 g), neomycin (1 g) or a combination of both antibiotics for 10 days	Combination of rifaximin and neomycin effective for CH_4_ elimination (87 vs. 33% neomycin vs. 28% rifaximin)	Combination of rifaximin and neomycin effective in improving clinical response (85 vs. 63% neomycin vs. 56% rifaximin)
	Pimentel et al. (2014)	RCT, CH_4_+ IBS-C patients (*n* = 32), Rome II	Neomycin (1 g) plus placebo (not specified) or in combination with rifaximin (1.65 g) for 14 days	Combination of neomycin and rifaximin reduced CH_4_ levels (10/15 subjects). Low levels of CH_4_ at baseline were associated with a greater improvement in constipation	Combination of rifaximin and neomycin superior in improving IBS-C symptoms
	Sharara et al. (2006)	RCT, IBS patients (*n* = 124), Rome II	Rifaximin (400 mg) versus placebo for 10 days, followed by 10-day washout period	Rifaximin responders reduced H_2_-breath excretion, which correlated with symptoms	Relief of general IBS symptoms (40.5% rifaximin vs. 18.2%), maintained during the washout period in some patients (27%)
	Li et al. (2020)	Prospective and open label trial, IBS-D patients (*n* = 30), Rome III Healthy individuals (*n* = 19)	Rifaximin (1.2 g) for 2 weeks	Changes in composition of faecal bacteria (*↑Bifidobacterium,* ↓*E. coli* and *Enterobacter*), rectal mucosal bacteria and faecal mycobiota, and certain metabolic pathways. Faecal bacterial dysbiosis identified as a tool to predict response to rifaximin	Abdominal symptoms more attenuated in IBS-D patients presenting with faecal bacterial signatures, more different from those of healthy individuals
	Rezaie et al. (2019)	Open label, IBS-D patients (*n* = 98), Rome III	Rifaximin (1.65 g) for 2 weeks, followed by a 4-week washout period	Normalisation of a positive LBT after rifaximin treatment, which was likely to predict response	Response to rifaximin in 48% of patients, with no symptom recurrence in 15.6% of them. Response rate higher in patients with normalised LBT post-treatment (76.5%)
	Soldi et al. (2015)	Open label, IBS-nonC patients (*n* = 15), Rome II Healthy individuals (*n* = 5)	Rifaximin (1.65 g) for 2 weeks, followed by a 6-week washout period Healthy individuals did not take any drug	Moderate changes in gut microbiota composition: stable overall profile, ↓*Clostridium* spp., ↑*Faecalibacterium prausnitzii*	Relief of global IBS symptoms in most of patients (80%). Of them, the majority had −LBT and improved overall IBS symptoms at end of study (73% after treatment and 78% washout period)
	Zhuang et al. (2018)	Open label, IBS-D (*n* = 30), Rome III Healthy individuals (*n* = 13)	Rifaximin (800 mg) for 2 weeks, followed by 10-week washout period Healthy individuals did not take any drug	Modulation of certain bacterial taxa: ↓Firmicutes and *Clostridiales,* ↑ *Bacteroidetes* and *Bacteroidales.* Potential changes in microbial metabolism: ↓propanoate and butanoate	General IBS symptoms improved after treatment and relief maintained at least 10 weeks. Out of 14 patients with SIBO, 9 had −LBT at day 28

Abbreviations: CH_4_, methane; CH_4_+, positive-methane; H_2_, hydrogen; IBS, irritable bowel syndrome; IBS-C, irritable bowel syndrome with constipation; IBS-D, irritable bowel syndrome with diarrhoea; IBS-nonC, irritable bowel syndrome without constipation (=diarrhoea and mixed bowel habits); LBT, lactulose breath test (methane and hydrogen excretion); L(H)BT, lactulose (hydrogen) breath test; *n*, size of population (selected or randomised patients depending on study intervention); QoL, quality of life; RCT, randomised controlled trial; SCFA, short-chain fatty acid; SIBO, small intestinal bacterial overgrowth. % indicates the percentage of patients or cases. Symbols*:* ↑, increase; ↓, decrease, +, positive; −, negative.

a Statistically significant findings unless otherwise specified.

b Reported total daily dose.

## Probiotics

Probiotics [~for life] are defined as live microorganisms that confer a health benefit on the host when administered in adequate amounts (Gibson et al., 2017). The use of probiotics for the management of GI symptoms gained popularity during the 1990s (Rolfe, 2000). Here we focus on placebo-controlled studies that have investigated the effect of probiotics on gut microenvironment and their interaction with IBS symptoms. To date, studies assessing the effects of probiotics on IBS symptoms as well as published meta-analyses provide mixed results regarding the clinical efficacy of probiotic products in IBS (Ford et al., 2018; McFarland et al., 2021).

### Bifidobacterium spp. and Lactobacillus spp.

Bifidobacteria is generally associated with health benefits throughout life, although its abundance varies with age (O’Callaghan and van Sinderen, 2016). Low levels of bifidobacteria in IBS (Pittayanon et al., 2019) may be improved after administration of probiotic bifidobacteria and successfully alleviate symptoms (Ford et al., 2018; Pinto-Sanchez et al., 2017). However, the link between the clinical benefit and the impact on the gut microenvironment after bifidobacteria supplementation needs further investigations (Table 2).

Box 3. Prebiotics – Key points

- The clinical benefit is dependent on prebiotic type and dose.
- Suggested mechanisms of action of prebiotics include increase of growth and activity/function of health-associated bacteria.
- Subgroup analyses and assessments of the effect of different doses are required to better understand the therapeutic potential of prebiotics in IBS.

**Table 2.** Overview of studies evaluating the effects of probiotics on the gut microenvironment and clinical outcome in patients with IBS.

Probiotic strain	References	Design, study cohort (*n*), Rome criteria	Intervention	Main findings: gut microenvironment^a^	Main findings: symptoms^a^	
*Bifidobacterium* spp.	Charbonneau et al. (2013)	RCT, IBS patients (*n* = 76), Rome II Healthy individuals (*n* = 41)	Encapsulated *B. infantis* 35624 (10^9^ CFU^b^) versus placebo for 8 weeks, and 2-week washout	Transient colonisation with *B. infantis* and limited effect on targeted microbiota profile	No impact on symptoms	
	Pinto-Sanchez et al. (2017)	RCT, IBS-nonC patients (*n* = 44), Rome III	*B. longum* NCC3001 (10^10^ CFU per sachet) versus placebo for 6 weeks, and 4-week washout	Reduction of methylamines and aromatic amino acids metabolites in urine. Changes in faecal microbiota profiles, serum inflammation markers and levels of neurotrophins and neurotransmitters were independent of probiotic	Improved depression scores (64% probiotic group vs. 32% placebo) and induced changes in brain activity	
*Lactobacillus* spp.	Cremon et al. (2018)	RCT, IBS patients (*n* = 42), Rome III	Crossover study, *L. paracasei* CNCM I-1572 (4.8·10^10^ CFU) versus placebo for 4 weeks, followed by 4-week washout	Modulation of microbiota composition (↓*Ruminococcus* genus; ↑*Lactobacillus*); microbiota function (↑acetate and butyrate); and proinflammatory cytokines (↓IL-15)	Not proven to improve IBS symptoms. For example, abdominal pain/discomfort: 37.5% probiotic group vs. 30% placebo, *p* > 0.05	
	Shin et al. (2018)	RCT, IBS-D patients (*n* = 60), Rome III	*L. gasseri* BNR17 (4·10^10^ CFU) versus placebo for 8 weeks	*L. gasseri* modulated certain bacterial taxa: ↑Actinobacteria, *Bifidobacterium* and *Lactobacillus*; ↓Proteobacteria, *Blautia* and *Faecalibacterium*	Symptom improvement in both active treatment and placebo. Higher improvement of generalised symptoms and QoL in probiotic group	
	Lewis et al. (2020)	RCT, IBS patients (*n* = 285), Rome III	*L. paracasei* versus *B. longum* (10^9^ CFU) versus placebo for 8 weeks	*L. paracasei* and *B. longum* detected in probiotic group but no changes throughout intervention, except for some patients without bifido at baseline. No changes in the relative abundance of *A. muciniphila* and *F. prausnitzii*	Both active and placebo groups reduced symptom severity, *L. paracasei* beneficial for bowel movements in IBS-C and IBS-D patients. *B. longum* ameliorated spontaneous bowel movements in IBS-D and IBS-C. Both probiotics beneficial for QoL	
	Murakami et al. (2012)	RCT, Rome III, IBS patients (*n* = 35)	Crossover study, *L. brevis KB290* (≥10^10^ CFU) versus placebo for 4 weeks each treatment with 4-week washout period in between	Shifts in some of the targeted bacterial taxa such as ↑*Bifidobacterium* and ↓*Clostridium*	No differences in IBS symptoms except for reduction of frequencies of watery stools and abdominal pain as well as improved QoL during the active treatment	
*Clostridium* spp.	Sun et al. (2018)	RCT, IBS-D patients (*n* =200), Rome III	*Clostridium butyricum* (5.67∙10^7^ CFU) versus placebo for 4 weeks	After intervention, microbial community more dissimilar between the groups. *Clostridium sensu stricto* reduced in responders of the active group. *C. butyricum* was predicted to be involved in amino acid, fatty acid and tryptophan metabolisms	Decrease in overall IBS symptoms, specifically frequency of bowel habits and QoL. Patients with moderate and severe symptoms at baseline benefited more from intervention with active treatment
Mixtures	Bonfrate et al. (2020)	RCT, IBS patients (*n* = 25), Rome IV	Crossover study, *B. longum* BB536 and *L. rhamnosus* HN001 (5∙10^9^ CFU) and vitamin B6 (LBB) versus placebo for 30 days each treatment with 15-day washout period	Amelioration of colonic permeability in some patients and shift in presumptive lactic acid bacteria. Some changes in faecal metabolites, for example, propanoic and butanoic acids	Decrease in IBS symptom severity, improved abdominal pain and bloating and relieved bowel habits and QoL
	Lyra et al. (2010)	RCT, IBS patients (*n* = 42), Rome II	*L. rhamnosus* GG*, L. rhamnosus* Lc705, *Propionibacterium freudenreichii* ssp. *shermanii* JS and *B. breve* Bb99 (8–9∙10^9^ CFU) for 6 months	Modulation of faecal bacterial phylotypes (↑*C. thermosuccinogenes* 85% and *R. torques* 93%, ↓ *R. torques* 94%)	Not investigated
	Yoon et al. (2015)	RCT, IBS patients (*n* = 81), Rome III	*L. acidophilus, L. rhamnosus, B. breve, B. actis, B. longum,* and *S. thermophilus* (10^10^ viable cells) versus placebo for 4 weeks	The active group changed probiotic bacteria (eg. ↑ *B. bifidum, B. lactis, L. acidophilus* and *L. rhamnosus*), but not the Firmicutes/Bacteroidetes ratio	The probiotic mixture tended to improve overall symptoms (74.4% prebiotic group vs. 61.9% placebo, *p* > 0.05) and was more effective in reducing diarrhoeal symptoms (*p* = 0.02)	
	Yoon et al. (2014)	RCT, IBS patients (*n* = 49), Rome III	*B. longum, B. bifidum, B. lactis, L acidophilus, L. rhamnosus*, and *S. thermophilus* (10^10^ viable cells) versus placebo for 4 weeks	The probiotic mixture induced alterations in the composition of intestinal microbiota, for example, ↑ *B. lactis, L. rhamnosus*, and *S. thermophilus* (active group) versus ↑*B. lactis* (placebo group)	The probiotic group improved IBS symptoms, including reduction of abdominal pain/discomfort and bloating intensity	
	Kajander et al. (2007)	RCT, IBS patients (*n* = 55), Rome I or II	*L. rhamnosus* GG, *L. rhamnosus* Lc705, *Propionibacterium freudenreichii* ssp. *shermanii* JS and *B. breve* Bb99 (9·10^9^ CFU) for 6 months	No changes in faecal microbiota and SCFAs after the intervention, but the bacterial enzyme ß-glucuronidase decreased	Not investigated	
	Ki Cha et al. (2012)	RCT, IBS-D patients (*n* = 50), Rome III	*L. acidophilus*, *L. plantarum*, *L. rhamnosus, B. breve*, *B. lactis, B. longum* and *S. thermophilus* (1·10^10^ CFU) versus placebo for 8 weeks, followed by 2-week washout period	Stabilisation of intestinal microbiota composition in the probiotics group (concordance rate of 69.5% active group vs. placebo 56.5%, *p* < 0.01)	Relief of overall IBS symptoms (48% probiotic group vs. 12% placebo), improvement of stool consistency, and a tendency towards improved IBS-QoL (*p* > 0.05)	
	Mezzasalma et al. (2016)	RCT, IBS-C patients (*n* = 150), Rome III	*L. acidophilus* and *L. reuteri* (4·10^9^ CFU) versus *L. plantarum, L. rhamnosus,* and *B. animalis* subsp. *lactis* (6·10^9^ CFU) versus placebo for 2 months, followed by 1-month washout period	Probiotic bacteria, except *B. animalis* subsp. *Lactis,* increased in the active group during the intervention with lasting effect of at least 90 days	Most patients reduced IBS-C related symptoms for at least 30 days, which correlated to improvement of QoL. The frequency of bowel movements normalised and stabilised over time
	Hod et al. (2018)	RCT, IBS-D patients (*n* = 109, only women), Rome III	*L. rhamnosus* LR5, *L. casei* LC5, *L. paracasei* LPC5, *L. plantarum* LP3, *L. acidophilus* LA, *B. bifidum* BF3, *B. longum* BG7, *B. breve* BR3*, B. infantis* BT1, *S. thermophilus ST3*, *L. bulgaricus LG1;* and *Lc. lactis* SL6 (5·10^10^ active bacteria) for 8 weeks	Stable microbial diversity with ↑ *Lactobacillus.* Responders: ↓ *Bilophila* proportion, changes in HS-CRP and faecal calprotectin. At baseline, higher proportions of *Faecalibacterium*, *Leuconostoc* and *Odoribacter* may predict beneficial inflammatory‐marker changes (*p* < 0.05)	Not investigated

Abbreviations: *B, Bifidobacterium; C*, *Clostridium*; CFU, colony-forming units; HS‐CRP high sensitivity C-Reactive Protein; IBS, irritable bowel syndrome; IBS-C, irritable bowel syndrome with constipation; IBS-D, irritable bowel syndrome with diarrhoea; QoL, quality of life; *L.*, *Lactobacillus; Lc, Lactococcus*; RCT, randomised controlled trial; *S.*, *Streptococcus.* % indicates the percentage of patients or cases. Symbols*:* ↑, increase; ↓, decrease.

a Statistically significant findings unless otherwise specified.

b Reported total daily dose.

Hitherto, there is no consensus concerning the potential role of *Lactobacillus* in IBS patients (Liu et al., 2017; Pittayanon et al., 2019). Still, the probiotic *Lactobacillus* is generally considered beneficial (Heeney et al., 2018) and its supplementation in IBS patients seems promising for managing their symptoms (Ford et al., 2018; Murakami et al., 2012). It has been suggested that *Lactobacillus* modulates specific bacterial taxa (Cremon et al., 2018; Murakami et al., 2012), alters the levels of faecal SCFAs or pro-inflammatory cytokines (Cremon et al., 2018; Shin et al., 2018), and could potentially be associated with improvement of IBS symptoms (Table 2).

Box 4. Food and dietary habits – Key points

1. Modifying the dietary habits constitutes an accessible and, to a large extent, safe strategy for IBS management, even though more extreme diets may be unfavourable for gut microbiota composition and function.
2. Dietary habits impact and interact with the gut microbiota.
3. The existing literature suggests that the management of IBS symptoms through dietary interventions may potentially involve modulation of gut microbiota composition, microbial metabolites, immune activity and intestinal permeability.
4. Further studies evaluating the short- and long-term impact of dietary habits on the gut microenvironment are needed.

*Lactobacillus* and *Bifidobacterium* may differ in their effect on modulating microbiota composition (Table 2; Supplementary References: *Bifidobacterium* spp. and *Lactobacillus* spp.). Overall, bifidobacteria show a greater tendency towards improvement of global IBS symptoms and pain scores (Ford et al., 2018), although *lactobacilli* may have a similar efficacy (Lewis et al., 2020).

Box 5. FMT – Key points

1. FMT application in IBS is still constrained to clinical research due to limitations in safety and partly divergent efficacy data.
2. Donors with a favourable microbiota (ie. diverse and stable) seem to be important for FMT success.
3. The poor link between microbiota alterations and symptom improvements suggests the involvement of other mechanisms of action that are yet to be clarified.
4. FMT shows a potential as a therapy for IBS, however, optimization of the protocol (eg. delivery route, dose and frequency) is required to move forward with clinical usage.

### Other probiotic bacteria and mixtures

Although the clinical potential of other single-strain probiotic bacteria has been investigated, the potential mechanisms of action are still insufficiently investigated (Ford et al., 2018)*. Clostridium butyricum,* however, is one of very few exceptions. After 4-week supplementation, *Clostridium butyricum* shifts the microbiota composition, as well as potentially modulates the metabolic pathways of amino acids, fatty acids and tryptophan in patients with IBS. Further, this probiotic may provide a greater benefit to patients with moderate and severe symptoms, and may alleviate overall IBS symptoms (Sun et al., 2018).

Probiotic mixtures may possibly have better efficacy than single-strain probiotics in IBS patients (Ford et al., 2018). Multiple formulations containing different combinations of *Lactobacillus* and *Bifidobacterium* strains as well as other bacterial genera have been tested in patients with IBS. Such formulations seem to have multiple effects on gut microenvironment, that is, shifting the microbiota composition (Bonfrate et al., 2020; Lyra et al., 2010; Yoon et al., 2014, 2015), ameliorating the colonic permeability (Bonfrate et al., 2020), or changing the bacterial enzyme ß-glucuronidase activity (Kajander et al., 2007), although not all studies are in agreement (Kajander et al., 2007; Ki Cha et al., 2012; Table 2). These mixtures may be effective in improving IBS symptoms and the severity of the disorder (Bonfrate et al., 2020; Yoon et al., 2014, 2015; Supplementary References: Mixtures) in certain IBS subtypes (Hod et al., 2018; Ki Cha et al., 2012; Mezzasalma et al., 2016) In addition, the response to the probiotic formulation may be predicted by faecal bacterial patterns at baseline (Hod et al., 2018; Table 2). Further, probiotic mixtures might also have a long-term effect on symptoms or specific bacterial taxa even after the supplement removal (Mezzasalma et al., 2016), although little is known about these effects.

### Current recommendations

While all these results are encouraging, the quality of evidence for IBS management is still poor and the general recommendation regarding the use of probiotics in clinical practice guidelines is weak. This reality is reflected in the recommendations in recent guidelines. In general, large professional societies recommend against widespread routine use of probiotics or avoid recommending specific products due to the weak supporting literature. Still, it is acknowledged that probiotics may be useful in select patients and that patient preference is important in the final decision of treatment regimen (Lacy et al., 2021; Su et al., 2020; Vasant et al., 2021). For this reason, further research with a higher degree of consensus concerning study design and bacterial strains may help fill the current knowledge gaps and improve the characterisation of the mechanisms of action that have so far been suggested (Figure 2).

## Prebiotics

Prebiotics, unlike probiotics, are not microorganisms but substrates that are selectively metabolised by health-promoting microorganisms (Gibson et al., 2017). Prebiotics, directly or through cross-feeding, enhance the activity (eg. SCFAs production) or growth of health-associated bacteria (eg. bifidobacteria and lactobacilli), without aggravating adverse effects such as distension due to gas production (Gibson et al., 2017). Since the first definition of prebiotics in the 90s, studies assessing the direct effect of prebiotics in IBS patients have been limited (Table 3) and with sparse but still potential clinical benefits in IBS (Ford et al., 2018).

**Table 3.** Overview of studies evaluating the effects of prebiotics on the gut microenvironment and clinical outcome in patients with IBS.

Prebiotic	References	Design, study cohort (*n*), Rome criteria	Intervention	Main findings: gut microenvironment^a^	Main findings: symptoms^a^
Inulin type fructans (ITFs)	Hunter et al. (1999)	RCT, IBS patients (*n* = 21), –	Cross-over study, Oligofructose (6 g^b^) versus placebo for 4 weeks	No change in fasting breath hydrogen concentrations	No effect on symptom scores, faecal weight and pH or whole-gut transit time
	Azpiroz et al. (2017)	RCT, IBS patients with rectal hypersensitivity (*n* = 79), Rome III	scFOS (5 g) versus placebo for 4 weeks	↑ Bifidobacteria	Reduction of anxiety scores. Tendency towards improved rectal sensitivity in IBS-C (*p* = 0.051)
Galacto-oligosaccharides (GOS)	Silk et al. (2009)	RCT, IBS patients (*n* = 44), Rome II	Crossover study, β-GOS (3.5 or 7 g) versus placebo for 4 weeks	↑ Bifidobacteria at both doses	Low dose modified stool consistency, reduced flatulence, bloating, composite score of symptoms, and SGA, whereas high dose decreased SGA and anxiety scores
Human Milk Oligosaccharides (HMOs)	Iribarren et al. (2020)	RCT, IBS patients (*n* = 60), Rome IV	2′FL/LNnT (5 or 10 g) versus placebo for 4 weeks	↑ Bifidobacteria in high dose at week 4 (lost after 4-week washout)	No worsening of symptoms (Phase II, safety study)
	Iribarren et al. (2021)	RCT, IBS patients (*n* = 58), Rome IV	2′FL/LNnT (5 or 10 g) versus placebo for 4 weeks	Change in overall faecal, but not mucosal microbiota composition in high dose. Both doses: ↑faecal and mucosal bifidobacteria and modulated certain bacterial taxa. Modulation of faecal and plasma, but not urine, metabolite profiles, associated with bifidogenic effect	Not investigated

Abbreviations: FOS, fructooligosaccharide; GOS, galactooligosaccharide; HMO, Human Milk Oligosaccharide; 2′FL/LNnT, 4:1 mix of 2′-fucosyllactose, and lacto-N-neotetraose; IBS, irritable bowel syndrome; IBS-C, irritable bowel syndrome with constipation; IBS-D, irritable bowel syndrome with diarrhoea; QoL, quality of life; scFOS; short-chain fructooligosaccharide; SGA, subjective global assessment; RCT, randomised controlled trial. Symbols*:* ↑, increase; ↓, decrease.

a Statistically significant findings unless otherwise specified.

b Reported total daily dose.

### Inulin type fructans

Inulin type fructans (ITFs) is a combined term for inulin, fructooligosaccharides (FOS) and oligofructose and refers to non-digestible, linear fructans, which can be found naturally in vegetables and fruits (Wilson and Whelan, 2017). Hunter et al. (1999) assessed the therapeutic effect of oligofructose (6 g/day) in IBS patients, in a 4-week crossover study, finding no modifications on symptom severity or fasting breathe hydrogen concentrations. Further, while high levels of FOS intake (20 g/day) caused transient worsening of symptoms (Olesen and Gudmand-Hoyer, 2000), a lower dose of short-chain fructooligosaccharides (scFOS) (5 g/day) was well tolerated in IBS patients with rectal hypersensitivity and led to significantly increased faecal bifidobacteria counts and decreased anxiety scores (Azpiroz et al., 2017). In this study, however, improvement in symptom severity was similar to placebo (Azpiroz et al., 2017).

### Galactooligosaccharides

β-galactooligosaccharides (β-GOS) are non-digestible oligosaccharides synthesised from lactose using microbial β-galactosidases, and unlike other GOS naturally found in plants they are selectively fermented by bacteria (Wilson and Whelan, 2017). A crossover pilot trial by Silk et al. (2009) showed that 4-weeks of β-GOS treatment at both 3.5 and 7 g/day relieved symptoms and, dose dependently, increased faecal bifidobacteria. In this study, the lower dose led to decreased flatulence and bloating, while the higher dose resulted in increased bloating but improved anxiety scores (Silk et al., 2009).

### Human milk oligosaccharides

Human breast milk contains high concentrations of structurally diverse glycans, collectively referred to as human milk oligosaccharides (HMOs). HMOs are non-digestible oligosaccharides, that are selectively metabolised by bacteria (eg. *Bifidobacterium longum* subsp. *infantis),* and beneficially influence infant gut health, and are considered as the first prebiotics new-borns encounter (Bode, 2012). However, studies on the prebiotic activity and health benefit of HMOs in adults are few. Recently our group conducted two studies on the impact of 4:1 HMO mix containing 2′-O-fucosyllactose and lacto-N-neotetraose (2´FL/LNnT) in IBS patients (Iribarren et al., 2020, 2021). Our findings show that adult patients tolerate 2′FL/LNnT supplementation, regardless of the dose (5 or 10 g/day) (Iribarren et al., 2020). Further, a potential change of the gut microenvironment was supported by modulation of the gut microbiota composition (Iribarren et al., 2020, 2021) and faecal and plasma metabolite profiles (Iribarren et al., 2021). When administered at 5 g/day for 12 weeks, the same 2′FL/LNnT formulation led to normalisation of stool forms and improvements in abdominal pain, bloating, overall IBS and quality of life scores in all IBS subgroups in a multicentre, placebo uncontrolled, open label trial (Palsson et al., 2020). However, given the large placebo effect on IBS symptoms, a placebo-controlled study is essential to confirm the clinical efficacy of HMOs in IBS.

### Current recommendations

According to the International Scientific Association for Probiotics and Prebiotics (ISAPP), ITFs and β-GOS are the only accepted prebiotics, while HMOs are promising candidates (Gibson et al., 2017). Overall, despite the bifidogenic effect reported in placebo-controlled studies, prebiotics do not seem to convincingly improve IBS symptoms or quality of life in IBS patients (Wilson et al., 2019; Table 3). Furthermore, due to high fermentability followed by gas production, high doses of prebiotics entail a risk to exacerbate symptoms in IBS patients (Muir, 2019). Yet, given the specific mechanistic effect of each prebiotic type, subgroup analyses as well as assessments of different doses of prebiotics may provide a more optimistic view of their potential to improve symptoms in IBS patients.

## Food and dietary habits

A large majority of IBS patients report generation and aggravation of GI symptoms following meal ingestion (Böhn et al., 2013). These symptoms are potentially triggered via primary (eg. prebiotic and osmotic effects) or secondary effects (eg. intraluminal pH and microbiome effects) (Spencer et al., 2014). Furthermore, local allergy-like reactions in the gut have recently been proposed to be of importance in subsets of patients (Aguilera-Lizarraga et al., 2021; Fritscher-Ravens et al., 2019). Patients commonly find food rich in carbohydrates and fat, dairy products or gluten as aggravating (Böhn et al., 2013). Thus, the exclusion or reduction of such food items from the diet is a regular practice to reduce symptoms (Lenhart et al., 2022), but comes with the risk of severe food avoidance and restriction in a proportion of IBS patients (Melchior et al., 2022). This habit may also negatively impact the gut microbiota (Altomare et al., 2021; Lenhart et al., 2022), reduce the quality of the diet (Altomare et al., 2021), and the overall nutrient intake (Tigchelaar et al., 2017). Hence, both positive and negative effects of dietary interventions and restrictions in IBS exist.

### Targeted carbohydrate reduction diet

Impaired absorption of short-chain (eg. fructose, sorbitol, lactose and sucrose) and long-chain (eg. starch) carbohydrates by the small intestine may contribute to GI symptoms (Hasler, 2006; Yao et al., 2014). The generation of symptoms may be explained by an altered microbial fermentation and carbohydrate metabolism (Yao et al., 2014) which can potentially be identified using breath tests (Gasbarrini et al., 2009; Table 4). Hence, the elimination or reduction of specific carbohydrates may improve IBS symptoms (Berg et al., 2013; Supplementary References: Targeted carbohydrate reduction) and modulate metabolomic pathways (Stenlund et al., 2021), but there is not yet any evidence for effect on inflammatory parameters (Nilholm et al., 2021). Nonetheless, clinical benefit may not always correlate with breath test results as a measure of microbial fermentation, so the exact mechanisms underlying symptom improvement with targeted carbohydrate reduction diets are still partly unresolved (Berg et al., 2013; Yao et al., 2014; Table 4).

**Table 4.** Overview of studies evaluating the effects of food on the gut microenvironment and clinical outcome in patients with IBS.

Diet	References	Design, study cohort (*n*), Rome criteria	Intervention	Main findings: gut microenvironment^a^	Main findings: symptoms^a^
Targeted carbohydrate reduction	Berg et al. (2013)	Open RCT, IBS patients (*n* = 202), Rome II	IBS diet with fructose-reduced diet versus IBS diet for 4 weeks	The effects of fructose-reduced diet were independent from fructose breath test result	Improvement of symptom scores and moderate changes of stool consistency after fructose-reduced diet
	Stenlund et al. (2021)	Open RCT, IBS patients (*n* = 105), Rome IV	Starch- and sucrose-reduced diet versus no dietary advice for 4 weeks	Reduction of starch intake, increased polyunsaturated fat; and metabolic effects mainly related to linoleic acid metabolism, fatty acid biosynthesis, and β-oxidation, but no correlations with symptoms	The starch- and sucrose-reduced diet improved bowel symptoms
	Laatikainen et al. (2020)	RCT, IBS patients (*n* = 23) and other functional GI disorders (*n* = 18), Rome IV	Crossover study. Ordinary versus hydrolysed high-protein, lactose-free milkshakes to be consumed for 10 days with 10-day washout period between interventions	No changes in inflammatory markers (TNF-α, IL-6), intestinal permeability (FABP2) or immune activation (1-methylhistamine)	Reduction of IBS symptoms severity and scores, for example, ↓ flatulence and heartburn
	Nilholm et al. (2021)	Open RCT, IBS patients (*n* = 105), Rome IV Non-IBS controls (*n* = 105)	Starch- and sucrose-reduced diet versus no dietary advice for 4 weeks	No changes in inflammatory cytokines	Improvement of several GI symptoms and influence on daily life scores after starch- and sucrose-reduced diet
Low FODMAPs	Bennet et al. (2018)	RCT, IBS patients (*n* = 67), Rome III	Low FODMAP diet versus Traditional IBS diet for 4 weeks	The low FODMAP diet impacted the faecal microbiota, for example, ↓bifidobacteria abundance. Faecal bacterial profiles predicted clinical response to low FODMAP intervention	Not investigated
	Hustoft et al. (2017)	RCT, IBS-D and IBS-M patients (*n* = 20), Rome III	Low FODMAP diet for 9 weeks. After 3 weeks, crossover challenge: FOS (~high FODMAP diet) versus placebo for 10 days, followed by 3-week washout period in between	The low FODMAP diet modulated proinflammatory cytokines, microbiota profile, and faecal SCFAs, that is, ↓IL-6 and IL-8, ↓*Faecalibacterium prausnitzii*, and *Bifidobacterium,* and ↓total SCFAs and *n*-butyric acid	The low FODMAP diet improved IBS symptoms, with higher alleviation rate under placebo challenge (80% placebo vs. 30% FOS)
	McIntosh et al. (2017)	Single blind RCT, IBS patients (*n* = 40), Rome III	Low versus high FODMAP diet for 3 weeks	Induced changes in the urine metabolome and H_2_ production; ↓Histamine; ↑Actinobacteria richness and diversity	IBS severity decreased in the low FODMAP group, with greater proportion of responders (72% low FODMAP diet vs. 21% high FODMAP diet)
	Valeur et al. (2016)	Intervention study, IBS patients (*n* = 63), Rome III	FODMAP restricted diet for 4 weeks	The low FODMAP diet changed SCFAs (↓acetic and *n*-butyric, or ↑i-butyric) and increased faecal proteolytic fermentation, regardless of IBS symptoms	Improvement of IBS severity
	Valeur et al. (2018)	Intervention study, IBS patients (*n* = 63), Rome III	FODMAP restricted diet for 4 weeks	Distinct effect on certain bacterial taxa depending on clinical response. Microbiota profile at baseline predicted response to the FODMAP restricted diet	Reduction of IBS-SSS scores
	Valdez-Palomares et al. (2021)	Prospective trial, IBS patients (*n* = 63), Rome III	Low FODMAP diet for 4 weeks	Response to the low FODMAP diet predicted at baseline with an accuracy of 96.87% based on faecal strains belonging to *Veillonella*, *Butyrivibrio* and *Ruminiclostridium*	68.75% responders versus non-responders 31.25%
	Vervier et al. (2021)	Prospective study, IBS-D and IBS-M patients (*n* = 56), Rome IV Household controls (*n* = 56 pairs)	Low FODMAP diet for 4 weeks, followed by 12 weeks of FODMAP rechallenge if symptoms improved, or usual diet if no improvement and for household control	The low FODMAP diet impacted IBS patients with pathogenic-like microbiota profile at baseline towards a health-like profile, for example, ↑Bacteroidetes, ↓ *Firmicutes* sp., and normalised primary metabolic genes	The low FODMAP diet had greater benefit on IBS symptoms in patients with a pathogenic-like microbiota profile (ΔIBS-SSS in pathogenic-like IBS *n* = 194 vs. healthy-like IBS *n* = 114)
	Eetemadi and Tagkopoulos (2021)	Compilation of microbiota and faecal metagenomics datasets, IBS patients (*n* = 152) Healthy subjects (*n* = 37)	Low FODMAP diet	Impact on gut microbiome. Defined potential benefit by high colonic CH_4,_ and SCFA production; and *Ruminococcus* 1, Ruminococcaceae UCG-002 and Anaerostipes	Not investigated
	Zhang et al. (2021)	RCT, IBS-D patients (*n* = 108), Rome III	Low FODMAP diet versus traditional dietary advice	The low FODMAP diet impacted faecal microbiota (↓*Bifidobacterium*) and faecal fermentation (↓ saccharolytic fermentation). Severe symptoms and high faecal fermentation index (saccharolytic fermentation) at baseline predicted response to low FODMAP diet	The low FODMAP diet provided an earlier relief in stool frequency and excessive wind, while overall symptom reduction was similar between groups (response rate: 55.6% low FODMAP vs. 48.1% traditional diet)
Gluten-free	Wu et al. (2017)	RCT, IBS-D patients (*n* = 28), Rome II	Gluten-free diet versus Gluten containing diet for 4 weeks	Changes in genes associated with intestinal permeability after gluten challenge	Not investigated
	Naseri et al. (2021)	Open RCT, IBS patients (*n* = 42), Rome IV	Open study. Combination of Low FODMAP and gluten-free diets for 6 weeks	Faecal microbiota modulation, for example, ↑*Bacteroidetes*, ↓Firmicutes/Bacteroidetes ratio; ↓ faecal calprotectin	Improvement in IBS symptoms severity in 73.3% of the patients

Abbreviations: CH_4_, methane; FABP2, Fatty Acid Binding Protein 2; FODMAP, Fermentable, Oligosaccharides, Disaccharides, Mono-saccharides, And Polyols; FOS, fructo-oligosaccharides; GI, gastrointestinal; H_2_, hydrogen; IL, interleukin; IBS, irritable bowel syndrome; IBS-C, irritable bowel syndrome with constipation; IBS-D, irritable bowel syndrome with diarrhoea; IBS-M, irritable bowel syndrome with mixed bowel habits; IBS-SSS, irritable bowel syndrome-severity score system; RCT, randomised controlled trial; SCFA, short-chain fatty acid; TNF-α, tumour necrosis factor alpha. % indicates the percentage of patients or cases. Symbols*:* ↑, increase; ↓, decrease.

a Statistically significant findings unless otherwise specified.

### Low FODMAP diet

The term Fermentable Oligo-, Di- and Mono-saccharides And Polyols (FODMAPs) was introduced by Gibson and Shepherd (2005). These dietary FODMAPs are poorly absorbed short-chain carbohydrates that reach the distal parts of the small intestine and the large intestine intact (Gibson and Shepherd, 2005). There, they constitute a source of nutrients for specific microbial species (McIntosh et al., 2017) and are fermented. As a result, gases, including hydrogen, methane and carbon dioxide, may be produced which together with increased intestinal water content via osmotic effects of the luminal carbohydrates, cause intestinal distension triggering GI symptoms. Therefore, reduction of foods rich in certain short-chain carbohydrates intake can impact hydrogen and methane production and luminal distension (Staudacher and Whelan, 2017).

Since the first retrospective study investigating the potential of a low FODMAP diet in patients with IBS and fructose malabsorption (Shepherd and Gibson, 2006), multiple studies have followed and increased the evidence-base for efficacy of the low FODMAP diet as a strategy for managing IBS (Black et al., 2021; Supplementary References: Low FODMAP diet). The positive symptomatic effect of reducing these carbohydrates may, however, in parallel negatively influence the colonic luminal microenvironment by altering gut microbiota composition and reduce bifidobacteria abundance, as well as other health-associated gut bacteria (Bennet et al., 2018; McIntosh et al., 2017; Table 4). Interestingly, the clinical response to a low FODMAP diet may be predicted by gut microbiota composition before the start of the dietary intervention (Bennet et al., 2018; Valdez-Palomares et al., 2021; Valeur et al., 2018), by high colonic methane and SCFA production (Eetemadi and Tagkopoulos, 2021), and by high saccharolytic fermentation activity (Zhang et al., 2021), supporting the importance of the gut microenvironment. However, not all studies have demonstrated a correlation between fermentation or gut microbiota profile and IBS symptoms (Table 4) or show substantial benefits in symptomatology (Nordin et al., 2022). In addition, even though a low FODMAP diet or β-GOS supplementation for 4 weeks have very similar effects on symptom improvement, discontinuation of a low FODMAP diet may lead to a faster reappearance of symptoms as compared to elimination of prebiotic β-GOS supplementation. This suggests that the effect of a low FODMAP diet on symptoms may be mediated through the food and its interactions with the microbiota, rather than a direct effect on microbiota composition (Huaman et al., 2018; Simrén, 2018).

The long-term efficacy and safety of the reduction of FODMAP intake are still surrounded by uncertainties. Even so, a low FODMAP diet seems to have a sustained effect on IBS symptoms and quality of life over at least 6 months and with a high rate of adherence (Supplementary References: Long-term low FODMAP diet). Nevertheless, to date, a long-term restrictive low FODMAP diet is not considered to be beneficial because it can result in nutritional deficiencies (Harvie et al., 2017) and it can negatively influence the gut microenvironment (Bennet et al., 2018; McIntosh et al., 2017). Although few long-term studies exist, two studies support that a modified low FODMAP diet can be nutritionally adequate up to 18 months, without adversely impacting on food-related quality of life (O’Keeffe et al., 2018), but may reduce diet quality (Staudacher et al., 2020). Consequently, following a period of strict FODMAP exclusion, a gradual reintroduction of selected FODMAP is recommended to identify tolerable long-term solutions and increase dietary variety and nutrients (Harvie et al., 2017).

### Gluten/wheat-free diet

Gluten is a complex, water-soluble protein found in grains. Following gluten intake, some IBS patients may report GI and extra-intestinal symptoms such as fatigue, similar to patients with coeliac disease, even though intestinal structural alterations are absent (Biesiekierski et al., 2011). This entity is known as non-coeliac gluten/wheat intolerance and can overlap with IBS (Spencer et al., 2014). The exclusion of gluten from the diet has been shown to successfully improve IBS symptoms in subgroups of patients (Supplementary References: Gluten-free diet), but not all studies agree with this (Nordin et al., 2022). Interestingly, one study demonstrated that approximately one third of IBS patients who benefit from a gluten-free diet suffer from wheat sensitivity (Barmeyer et al., 2017), which may be triggered by high levels of the FODMAP fructan found in wheat (Spencer et al., 2014). The relevance of fructan, rather than gluten, for symptom generation in these patients was also supported by a recent randomised controlled challenge study (Skodje et al., 2018). Hence, gluten may not be the unique offensive factor (Biesiekierski et al., 2013) and it is currently often wheat, rather than gluten *per se*, that is considered to be linked to IBS (Zannini and Arendt, 2018).

So far, the vast knowledge about the effect of gluten/wheat restricted diets on the gut microenvironment is obtained by studies on healthy individuals. These studies have described the ability of a gluten-free diet to shape the gut microbiota composition (Bonder et al., 2016; De Palma et al., 2009; Hansen et al., 2018; Pinto-Sanchez et al., 2020) and modulate activity levels of bacterial metabolic pathways (Bonder et al., 2016). Additionally, a low gluten diet seems to induce moderate changes in the gut microbiota (eg. decreasing bifidobacteria), microbial function and host physiology biomarkers in healthy individuals. All these changes are thought to lead to relative improvements of GI symptoms (eg. bloating) although the role of gluten *per se* is still uncertain (Hansen et al., 2018). On the other hand, studies conducted on patients with coeliac disease show that strict gluten restriction may lead to an unbalanced diet (eg. high sugar, high fat and low fibre) and create a high risk for nutritional deficiencies (eg. calcium and iron) raising concerns regarding potential effects of long-term adherence (Bardella et al., 2000; Kinsey et al., 2008; Wild et al., 2010). To the best of our knowledge, the nutritional challenges of a gluten-free diet have not yet been studied in IBS and its effect on the gut microenvironment is poorly understood. A gluten-free diet possibly improves bowel habits (Vazquez-Roque et al., 2013) and modulates intestinal permeability (Vazquez-Roque et al., 2013; Wu et al., 2017), and a diet low in both gluten and FODMAP has been suggested to improve IBS symptoms severity while normalising the gut microbiota composition in IBS patients (Naseri et al., 2021), but more studies are certainly needed on this topic in IBS (Table 4).

### Current recommendations

To conclude, exclusion of certain food items seems to relieve symptoms in a large proportion of IBS patients, at least in the short term. Moreover, professional dietary guidance can prevent avoidance of food items crucial for health (McKenzie et al., 2016) and improve quality of life and diet (Ostgaard et al., 2012). For the moment, the current guidelines carefully include the diet low in FODMAPs as a second-line dietary therapy for IBS management, because of its invasiveness, safety concerns and the low-quality evidence to support its long-term use to alleviate global IBS symptoms, even though the proof of its efficacy in the short term is good. Moreover, the low FODMAP diet is strict and requires guidance by a dietician. Due to the lack of understanding of long-term effects on nutritional adequacy and gut health, its implementation should be short-term (Moayyedi et al., 2019; van Lanen et al., 2021; Vasant et al., 2021), and thereafter a gradual reintroduction of FODMAPs is recommended (Whelan et al., 2018). The effect of a gluten-free diet, however, is unclear and there is consequently a recommendation against its widespread use in IBS. Dietary recommendations concerning other diets cannot be made due to lack of objective information. Food can be regarded as the crossroads between pathogenesis, symptom origin and symptom control (Spencer et al., 2014), and the importance of this intersection is expected to attract a lot of attention in the coming years.

## Faecal microbiota transplantation

FMT is a 1,700-year-old, unconventional therapy (Zhang et al., 2012), which aims to favourably alter gut microbiota composition through administration of faecal material from healthy individuals into the GI tract of patients with presumed gut microbiota alterations. First applied to treat food poisoning and severe diarrhoea in the fourth century (Zhang et al., 2012) and now effectively used for treatment of *Clostridium difficile* infection, FMT has produced varying outcomes in IBS patients in regards to symptoms and gut microenvironment in research settings (König et al., 2017).

**Figure 3:**
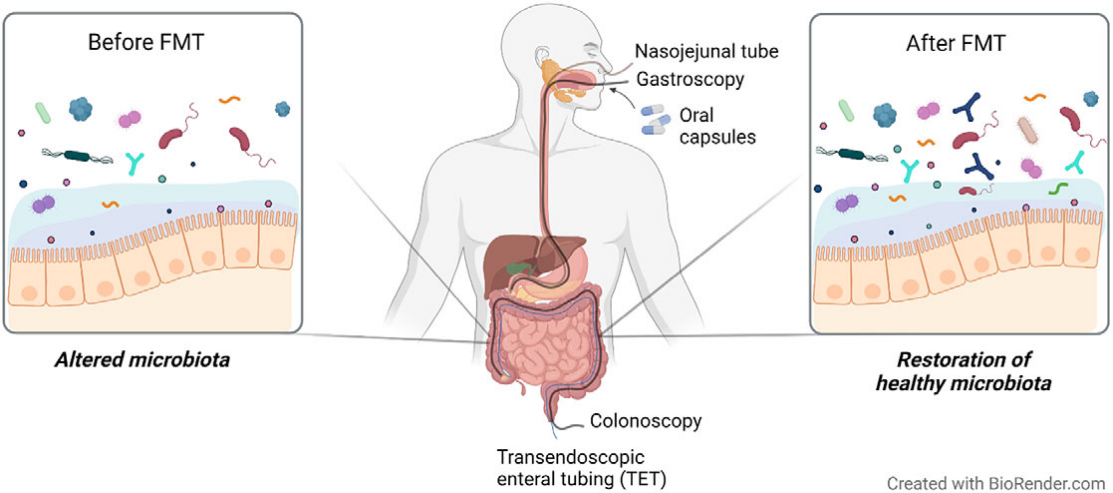
Faecal microbiota transplantation (FMT) as a strategy to restore healthy gut microbiota in IBS. Overall, FMT aims to shift the altered microbiota towards homeostasis through colonisation of healthy donor microbiota. The processed faecal material obtained from a healthy donor can be administered through upper (eg. oral capsules, gastroscopy and nasojejunal tube) or lower [eg. colonoscopy and transendoscopic enteral tubing (TET)] GI routes. Oral capsules generally contain very small amounts of faecal material and require multiple ingestions daily. FMT delivery through gastroscopy and nasojejunal route involves administration of a flexible tube through the mouth and nose, respectively, into the small intestine. In colonoscopy, FMT is delivered to the colon through a flexible tube inserted through the anus. The colonoscopy channel can also be used to introduce TET into the colon. The TET is then fixed to the mucosa using titanium clips for whole-colon FMT delivery.

Several strategies are currently available to perform a FMT (Figure 3) and may explain the diversity of outcomes described in patients with IBS (Table 5). One way to administer FMT is through oral capsules, which have been shown to change the gut microbiota to resemble that of the donors. However, microbiota alterations do not seem to translate well into clinical improvement and contrary to what was expected, FMT treatment may have less or comparable efficacy to placebo (Aroniadis et al., 2019; Halkjær et al., 2018). While this method is easy to administer and is cost effective, it may require the intake of multiple capsules a day, which can trigger feelings of nausea and vomiting (Gulati et al., 2020). Another approach is to deliver faecal material to the colon (Holster et al., 2019; Lahtinen et al., 2020), commonly performed via colonoscopy (Gulati et al., 2020). This method of FMT may shift the recipient’s microbiota (Lahtinen et al., 2020), but does not seem to increase butyrate-producing bacteria following treatment (Holster et al., 2019) and so far has not been demonstrated to be superior to the placebo group receiving autologous stool in improving symptom severity (Holster et al., 2019; Lahtinen et al., 2020). Furthermore, the high cost and requirement of pre-treatment (ie. bowel cleansing), that may influence symptoms and gut microenvironment, make it difficult to interpret the clinical outcome of colonoscopic FMT (Gulati et al., 2020; Holster et al., 2019). Alternatively, FMT delivered by transendoscopic enteral tubing (TET) does not require bowel preparation and is convenient for multiple administrations (Gulati et al., 2020). Delivery of microbiota isolates obtained from donor faeces using TET improved symptom severity and quality of life as well as changed the dominant microbial taxa in responders following FMT (Huang et al., 2019).

**Table 5.** Overview of studies evaluating the effects of faecal microbiota transplantation on the gut microenvironment and clinical outcome in patients with IBS.

Route of administration	References	Design, study cohort (*n*), Rome criteria	Intervention	Main findings: gut microenvironment^a^	Main findings: symptoms^a^
Oral	Halkjær et al. (2018)	RCT, IBS patients (*n* = 52), Rome III	25 FMT capsules/day containing 50 g of donor stool for 12 days versus placebo capsules	Increased faecal microbial biodiversity and shift towards donor microbiota	Placebo capsules more effective in improving IBS severity (79.2% response in placebo vs. 36.4% FMT) and QoL 3 months after treatment
	Aroniadis et al. (2019)	RCT, IBS-D patients (*n* = 48), Rome III	Crossover study (12-week interval), 25 FMT capsules/day containing 9.5 g for 3 days versus placebo capsules	Shift towards donor microbiota with no difference in microbial diversity and donor similarity between responders and non-responders. No change in bacterial abundance after FMT	Similar improvement in IBS severity and clinical response between FMT-first and placebo-first at 3-month post-treatment (Response rate 50 vs. 61%, respectively)
Colonoscopy	Holster et al. (2019)	RCT, IBS patients low in butyrate-producing bacteria (*n* = 17), Rome III	30 g of donor stool versus autologous stool	Similar microbial diversity and butyrate-producing bacteria abundance after FMT. Alterations in faecal and mucosa adherent bacterial composition in both groups	Reduction of symptom severity (from week 4) and improvement of QoL (from week 2) only in FMT. No difference between active treatment and placebo
	Lahtinen et al. (2020)	RCT, IBS-nonC patients (*n* = 49), Rome III	30 g of donor stool versus autologous stool	Shift towards donor microbiota, increased bacterial richness but similar microbial diversity in the FMT group compared to baseline	Decreased symptom severity in the FMT recipient 3 months post-treatment. No difference between active treatment and placebo. No effect on QoL, depression and anxiety
Transendoscopic enteral tubing (TET)	Huang et al. (2019)	Intervention study, refractory IBS patients (*n* = 30), −	Microbiota suspension from donor stool, 2–3 times every other day	IBS-D responders higher diversity at baseline. Change in dominant microbiota composition post-FMT in responders (IBS-D): *Methanobrevibacter* and *Akkermansia* most abundant bacteria post-FMT	Decreased symptom severity, and improvement of QoL at 1 month and 3-month post FMT
Gastroscopy	Mazzawi et al. (2019)	Intervention study, IBS-D patients (*n* = 13), Rome III	30 g of donor stool	↑SCFA and change in microbiota composition after FMT (more similar to that of the donors). Trend towards increased bacterial diversity in both responders and non-responders. Correlations between gut microenvironment and symptom severity	Clinical response in 62% of patients. Decreased symptom severity, increased QoL and improved stool consistency in responders
	El-Salhy et al. (2020)	RCT, IBS patients (*n* = 164), Rome IV	30 or 60 g of donor stool versus autologous stool	No change in the dysbiosis index. Change in bacterial abundance post-treatment; 30 g: ↑*Alistipes, Bacteriodes* and *Prevotella* spp. ↓*Eubacterium halii* and *Firmicutes* spp. 60 g: ↑*Alistipes* and *Akkermansia muciniphila* spp. ↓*Dorea* spp.	Reduction of symptom severity, fatigue and QoL amelioration in both FMT doses compared to placebo 3 months after treatment. Response rate of 30 g, 60 g and placebo; 76.9, 89.1 and 23.6%, respectively
	El-Salhy et al. (2021)	RCT, IBS patients (*n* = 142), Rome IV	30 or 60 g of donor stool versus autologous stool	Both groups increased faecal butyric acid. Increased total SCFA only in the 60 g group post FMT. Increase in butyric acid inversely correlated with symptom severity and fatigue in responders	Improvement of symptom severity, fatigue and QoL compared to placebo in both doses 1 month post FMT
Nasojejunal tube	(Holvoet et al., 2021)	RCT, refractory IBS-D or IBS-M with severe bloating (*n* = 62), Rome III	Crossover study, donor stool versus autologous stool	Higher microbial diversity and different overall microbial composition at baseline in responders	Ameliorated general IBS symptoms, abdominal bloating (56% response vs. 26% response in placebo), IBS symptom scores and QoL at 12 weeks post FMT

Abbreviations: FMT, faecal microbiota transplantation; IBS, irritable bowel syndrome; IBS-D, irritable bowel syndrome with diarrhoea; IBS-nonC, irritable bowel syndrome without constipation (= diarrhoea and mixed bowel habits); IBS-M, irritable bowel syndrome with mixed bowel habits; QoL, quality of life; RCT, randomised controlled trial; SCFA, short-chain fatty acid. Symbols*:* ↑, increase; ↓, decrease.

a Statistically significant findings unless otherwise specified.

Delivery of stool transplant through gastroscopy may normalise SCFA levels (El-Salhy et al., 2021; Mazzawi et al., 2019) and change gut microbiota composition (Cruz-Aguliar et al., 2019; Mazzawi et al., 2019), while improving symptoms and QoL in IBS patients (Cruz-Aguliar et al., 2019; El-Salhy et al., 2020; Mazzawi et al., 2019). Some of the changes in the gut microenvironment correlated with IBS symptom severity in the patients responding to the FMT treatment (Cruz-Aguliar et al., 2019; El-Salhy et al., 2021). Similar to gastroscopy, the use of nasojejunal tube to deliver FMT has led to promising results including improvements in general IBS symptoms, abdominal bloating, quality of life, and decrease in selected symptom scores in the active treatment group compared to the placebo group receiving autologous stool (Holvoet et al., 2021). Furthermore, responders had higher microbial diversity and different overall microbial composition at baseline compared with non-responders (Holvoet et al., 2021). Although costly, FMT delivery through gastroscopy and nasojejunal tube, are potentially less invasive than colonoscopy and can be performed without bowel cleansing (Gulati et al., 2020).

### Current recommendations

Considering the relatively benign nature of IBS, and the potential risks with FMT, the general concern has been whether it is worth the trade-off. While there is a call for caution (Camilleri, 2021), the adverse effects of FMT in IBS patients have been limited to short-term and self-limiting GI symptoms. As of now, FMT is not an accepted therapy for clinical use in IBS and should still be limited to clinical trials (König et al., 2017), even though recently published large controlled FMT trials provide hope for future use in selected patients (El-Salhy et al., 2020; Holvoet et al., 2021). Still, the efficacy is debatable, which could be attributed to differences in the methods used (Table 5). Overall, FMT delivery through gastroscopy or via a nasojejunal tube have been the most promising options and showed dose-dependent and long-term improvements in IBS patients (El-Salhy et al., 2020, 2022; Holvoet et al., 2021). The positive outcome can be attributed to the identification of a donor with well-defined favourable microbiota (diverse and stable over time) and recipients that are more likely to respond to the treatment (diverse and less disturbed microbiota) (El-Salhy et al., 2020; Holvoet et al., 2021). Overall, while these results are encouraging, data regarding the optimal FMT protocol (eg. delivery route, dose and frequency) and mechanisms underlying symptom improvement are yet to be established.

## Combined treatments

The treatment strategies described in this review have yielded some clinical benefits, but with mixed and sometimes inconsistent results regarding efficacy. To overcome the limitations of single treatment interventions, combinations of different strategies have been tested.

Restrictive dietary interventions, while improving symptoms, may paradoxically have adverse effects on the gut microenvironment. The low FODMAP diet, a well-recognised IBS therapy, has been shown to reduce faecal bifidobacterial abundances and butyrate as well as total SCFA levels, which otherwise are associated with health benefit (Huaman et al., 2018; Staudacher et al., 2017; Wilson et al., 2020). Conversely, prebiotics have a bifidogenic effect and have been suggested as a supplementary treatment for the low FODMAP diet to potentially overcome the negative effects on gut microbiota composition and function with this restrictive diet (Huaman et al., 2018). Interestingly, co-administration of a probiotic mixture containing *Bifidobacterium* spp., together with a low FODMAP diet, increased faecal *Bifidobacterium* and *Lactobacillus* abundances (Staudacher et al., 2017, 2021). However, supplementation of β-GOS during the low FODMAP diet improved symptoms, but did not lead to increase in bifidobacteria (Wilson et al., 2020). On the other hand, high levels of FOS (16 g/day), while increasing bifidobacteria, led to worsening of symptoms in IBS patients on a low FODMAP diet (Hustoft et al., 2017), highlighting the importance of the dose used.

Another strategy for combined therapy is to co-administer probiotics together with prebiotics, now referred to as complementary synbiotics. Synbiotics are “a mixture comprising live microorganisms and substrate(s) selectively utilised by host microorganisms that confers a health benefit on the host” (Swanson et al., 2020). According to this definition, synbiotics may also comprise live organisms and substrates that are not probiotics and prebiotics respectively, which in this case are referred to as synergistic synbiotics (Swanson et al., 2020). The delivery of synbiotic fermented milk, which included inulin (90 per cent) and oligofructose (10 per cent) as prebiotics together with the probiotics *Lactobacillus* and bifidobacteria, demonstrated transient increase in administered strains in faecal samples of IBS patients (Bogovič Matijašić et al., 2016). However, the effects of synbiotics on IBS symptoms vary across studies (Chlebicz-Wójcik and Śliżewska, 2021).

## Metabolites

Metabolites produced or modulated by intestinal bacteria have been suggested to play a role in IBS pathophysiology, either directly by chemical interactions or via local modulation of microbiota and/or immune activity (Rajilić-Stojanović et al., 2015). SCFAs and bile acids are two of the most targeted metabolites in IBS related research and regarded as potential treatment targets.

### Short-chain fatty acids

SCFAs, mainly acetate, propionate, and butyrate, are the most prominent by-products of colonic microbiota-mediated metabolism of indigestible dietary fibre. Their established role in intestinal homeostasis (Tan et al., 2014) and the recent suggestion of their involvement in microbiota-gut–brain interaction (Dalile et al., 2019) have attracted interest to study SCFAs in IBS pathophysiology. Some IBS patients may have lower levels of SCFA-producing bacteria (Pozuelo et al., 2015). Furthermore, a recent meta-analysis indicates globally lower SCFA levels, including butyrate, in IBS-C patients and higher SCFA levels in IBS-D patients (Sun et al., 2019). In line with this, it has been demonstrated that receiving microencapsulated sodium butyrate for 12 weeks, as a supplemental therapy, reduced the frequency of spontaneous and postprandial abdominal pain, the pain during defecation and constipation compared to placebo, while there were no significant effects on the severity of other GI symptoms (Banasiewicz et al., 2013). Furthermore, modulation of SCFAs, described in other gut microbiota-targeted therapies along with association of SCFAs to health-promoting bacteria, may serve as a potential marker linked to symptom improvement (see Tables 1–5).

### Bile acid metabolism

Bile acids are synthesised in the liver and released into the intestinal lumen to be mostly reabsorbed upon reaching the terminal ileum, while the remaining bile acids (<5 per cent) pass into the colon. Once in the colon, primary bile acids are modified by the bacteria via enzymatic processes, releasing secondary bile acids that mediate series of signalling events (Zhan et al., 2020). Approximately one third of the IBS-D patients, are reported to have bile acid malabsorption (BAM) (Wedlake et al., 2009), which leads to increased amounts of bile acids reaching the colon, where they can stimulate intestinal motility and secretion and potentially also exaggerate visceral hypersensitivity (Bajor et al., 2015; Dior et al., 2016; Wei et al., 2020). Bile acid sequestrants are part of the recommended therapy for diarrhoea in IBS patients despite limited supporting evidence from large randomised controlled trials. However, side effects and possible interference with other medications may make long-term use problematic (Nee et al., 2015). Several bacteria involved in bile acid metabolism seem to be altered in IBS patients (Zhan et al., 2020). Potentially *Clostridia* species, enriched in a subset of IBS-D patients, suppress the feedback-loop regulating bile acid synthesis through metabolites, further increasing colonic bile acid exposure (Zhao et al., 2020). Accordingly, as shown by a longitudinal study, the microbial biotransformation of bile acids is altered in both IBS-C and IBS-D patients, with IBS-D patients having higher and IBS-C patients having lower faecal primary bile acid levels compared to healthy individuals. Furthermore, the primary bile acids were increased in both IBS groups during flares (Mars et al., 2020). While changes in gut microbiota composition seem to play an important role in altered bile acid metabolism, the microbiota-bile acid axis is rarely assessed in studies on antibiotic, dietary, prebiotic and probiotic interventions. Indeed, the microbiota-bile acid interaction may be involved in explaining the effects of the aforementioned interventions on symptoms and could be a valid treatment target in future IBS trials.

### Current recommendations

Abnormalities in metabolite profiles may partially reflect affected biological pathways involved in IBS symptom generation, further reflected in metabolite alterations specific to each IBS subtype. Targeting the gut microenvironment through intervention studies (eg. antibiotics, probiotics, prebiotics and FMT), with thorough analysis of secondary effects on metabolites may be important for more effective long-term therapeutic strategies. Furthermore, metabolomic alterations, may also provide mechanistic insights into treatment response, as suggested by a post hoc analysis of a dietary intervention study (Nybacka et al., 2021). Overall, while the role of SCFA is extensively studied, the role of other metabolites, such as bile acids, neurotransmitters and vitamins, remains to be explored (James et al., 2020).

## Conclusion

IBS is a multifaceted disorder with a complex pathophysiology, where the gut microenvironment seems to play a key role in symptom generation. Evidence suggest that multiple luminal factors may be associated with IBS, including gut microbiota, microbial and dietary metabolites, and immune-related factors. Therefore, interventions targeting the gut microenvironment may be a coherent approach to indirectly provide health-related benefit to patients with IBS. This narrative review gathers the current knowledge on strategies that consider gut microenvironment modulation as a therapeutic target, with a specific focus on the link between effects in the gut microenvironment and impact on IBS symptoms.

Antibiotics, probiotics, prebiotics, food and FMT are strategies with potential to manage IBS symptoms (Figure 1). They vary in their effect on the gut microenvironment and present some limitations. For antibiotics, we largely lack mechanistic understanding on symptom improvement, especially regarding their effect on microbiota and how relevant this is for symptom improvement. The effect on the gut microenvironment may be highly dependent on dose and/or type of probiotics and prebiotics, and the link to symptom improvement also remains to be fully established. Certain dietary interventions seem to have favourable effects on symptoms, but long-term commitment may lead to undesired effects on the gut microenvironment, for example, a strict low-FODMAP diet reduces health-associated bacteria in the gut. The heterogeneity in study designs in FMT makes it difficult to draw conclusions on efficacy and the impact on the gut microenvironment, as well as mechanisms behind symptom improvement and factors of importance for donor selection. Still, combination of certain therapies might be an alternative approach, although solid scientific support for this approach is needed before being implemented in the clinic. In addition, a better understanding of the importance of metabolomic alterations reflecting host and microbial metabolism could help in developing more evidence-based therapies and predicting potential response in the individual patient.

To conclude, treatments targeting the gut microenvironment are promising, but improved knowledge regarding their specific mode of action, mechanisms underlying their effects on symptoms, the effect of different doses, is still needed to potentially be able to identify the right treatment to the right patient.

## Supplementary material

For supplementary material accompanying this paper visit https://doi.org/10.1017/gmb.2022.6. click here to view supplementary material
