# Supplementary material for: Modulating the gut microenvironment as a treatment strategy for irritable bowel syndrome: a narrative review
Source: Gut Microbiome (Camb). 2022 Aug 25;3:e7. doi: 10.1017/gmb.2022.6 (PMC11406401; doi:10.1017/gmb.2022.6)
Supplement: Supplementary file 1 [file S2632289722000068sup001.docx]

**Supplementary References:**

Studies evaluating the effects of antibiotics, probiotics, prebiotics and food and dietary habits in IBS symptoms and quality of life.

**ANTIBIOTICS**

*Rifaximin*

Lembo et al. (2016)

Lembo et al. (2020)

Pimentel et al. (2011)

Pimentel et al. (2006)

Cash et al. (2017)

**PROBIOTICS**

*Bifidobacterium* spp.

Ford et al. (2018)

Guglielmetti et al. (2011)

*Lactobacillus* spp.

Kim et al. (2018)

Ligaarden et al. (2010)

*Mixtures*

Kajander et al. (2005)

Ringel-Kulka et al. (2011)

**PREBIOTICS**

Niv et al. (2016)

**FOOD AND DIETARY HABITS**

Targeted carbohydrate reduction

Goldstein et al. (2000)

Vernia et al. (1995)

Saidi et al. (2021)

Austin et al. (2009)

Böhmer and Tuynman (2001)

Böhmer and Tuynman (1996)

Ong et al. (2010)

Low FODMAP diet

Ong et al. (2010)

de Roest et al. (2013)

Staudacher et al. (2011)

Staudacher et al. (2012)

Halmos et al. (2014)

Eswaran et al. (2016)

Böhn et al. (2015)

Zhang et al. (2021)

Zahedi et al. (2018)

Pedersen, Andersen, et al. (2014)

Pedersen, Vegh, et al. (2014)

Paduano et al. (2019)

Schumann et al. (2018)

Patcharatrakul et al. (2019)

Pirkola et al. (2018)

van Lanen et al. (2021)

Long-term efficacy of low FODMAP diet

O'Keeffe et al. (2018)

Harvie et al. (2017)

Weynants et al. (2020)

Maagaard et al. (2016)

Gluten/wheat-free diet

Zanwar et al. (2016)

Hajiani et al. (2019)

Barmeyer et al. (2017)

Paduano et al. (2019)

Pinto-Sanchez et al. (2020)

Shahbazkhani et al. (2015)

Peters et al. (2014)

Aziz et al. (2016)

Vazquez-Roque et al. (2013)

Barone et al. (2020)

**FAECAL MICROBIOTA TRANSPLANTATION**

Johnsen et al. (2018)

Holvoet et al. (2021)

Cui et al. (2021)

El-Salhy et al. (2021)

**REFERENCES**

**Austin, G. L., Dalton, C. B., Hu, Y., Morris, C. B., Hankins, J., Weinland, S. R., Westman, E. C., Yancy, W. S., Jr., and Drossman, D. A.** (2009). A very low-carbohydrate diet improves symptoms and quality of life in diarrhea-predominant irritable bowel syndrome. *Clinical Gastroenterology and Hepatology*, **7**(6), 706-708.e701. <https://doi.org/10.1016/j.cgh.2009.02.023>

**Aziz, I., Trott, N., Briggs, R., North, J. R., Hadjivassiliou, M., and Sanders, D. S.** (2016). Efficacy of a gluten-free diet in subjects with irritable bowel syndrome-diarrhea unaware of their HLA-DQ2/8 genotype. *Clinical Gastroenterology and Hepatology*, **14**(5), 696-703.e691. <https://doi.org/10.1016/j.cgh.2015.12.031>

**Barmeyer, C., Schumann, M., Meyer, T., Zielinski, C., Zuberbier, T., Siegmund, B., Schulzke, J. D., Daum, S., and Ullrich, R.** (2017). Long-term response to gluten-free diet as evidence for non-celiac wheat sensitivity in one third of patients with diarrhea-dominant and mixed-type irritable bowel syndrome. *International Journal of Colorectal Disease*, **32**(1), 29-39. <https://doi.org/10.1007/s00384-016-2663-x>

**Barone, M., Gemello, E., Viggiani, M. T., Cristofori, F., Renna, C., Iannone, A., Di Leo, A., and Francavilla, R.** (2020). Evaluation of non-celiac gluten sensitivity in patients with previous diagnosis of irritable bowel syndrome: a randomized double-blind placebo-controlled crossover trial. *Nutrients*, **12**(3). <https://doi.org/10.3390/nu12030705>

**Böhmer, C. J., and Tuynman, H. A.** (1996). The clinical relevance of lactose malabsorption in irritable bowel syndrome. *European Journal of Gastroenterology & Hepatology*, **8**(10), 1013-1016. <https://doi.org/10.1097/00042737-199610000-00015>

**Böhmer, C. J., and Tuynman, H. A.** (2001). The effect of a lactose-restricted diet in patients with a positive lactose tolerance test, earlier diagnosed as irritable bowel syndrome: a 5-year follow-up study. *European Journal of Gastroenterology & Hepatology*, **13**(8), 941-944. <https://doi.org/10.1097/00042737-200108000-00011>

**Böhn, L., Störsrud, S., Liljebo, T., Collin, L., Lindfors, P., Törnblom, H., and Simrén, M.** (2015). Diet low in FODMAPs reduces symptoms of irritable bowel syndrome as well as traditional dietary advice: a randomized controlled trial. *Gastroenterology*, **149**(6), 1399-1407.e1392. <https://doi.org/10.1053/j.gastro.2015.07.054>

**Cash, B. D., Pimentel, M., Rao, S. S. C., Weinstock, L., Chang, L., Heimanson, Z., and Lembo, A.** (2017). Repeat treatment with rifaximin improves irritable bowel syndrome-related quality of life: a secondary analysis of a randomized, double-blind, placebo-controlled trial. *Therapeutic Advances in Gastroenterology*, **10**(9), 689-699. <https://doi.org/10.1177/1756283x17726087>

**Cui, J., Lin, Z., Tian, H., Yang, B., Zhao, D., Ye, C., Li, N., Qin, H., and Chen, Q.** (2021). Long-term follow-up results of fecal microbiota transplantation for irritable bowel syndrome: a single-center, retrospective study [Original Research]. *Frontiers in Medicine*, **8**(1181). <https://doi.org/10.3389/fmed.2021.710452>

**de Roest, R. H., Dobbs, B. R., Chapman, B. A., Batman, B., O'Brien, L. A., Leeper, J. A., Hebblethwaite, C. R., and Gearry, R. B.** (2013). The low FODMAP diet improves gastrointestinal symptoms in patients with irritable bowel syndrome: a prospective study. *International Journal of Clinical Practice*, **67**(9), 895-903. <https://doi.org/10.1111/ijcp.12128>

**El-Salhy, M., Casen, C., Valeur, J., Hausken, T., and Hatlebakk, J. G.** (2021). Responses to faecal microbiota transplantation in female and male patients with irritable bowel syndrome. *World Journal of Gastroenterology*, **27**(18), 2219-2237. <https://doi.org/10.3748/wjg.v27.i18.2219>

**Eswaran, S. L., Chey, W. D., Han-Markey, T., Ball, S., and Jackson, K.** (2016). A randomized controlled trial comparing the low FODMAP diet vs. modified NICE guidelines in US adults with IBS-D. *The American Journal of Gastroenterology*, **111**(12), 1824-1832. <https://doi.org/10.1038/ajg.2016.434>

**Ford, A. C., Harris, L. A., Lacy, B. E., Quigley, E. M. M., and Moayyedi, P.** (2018). Systematic review with meta-analysis: the efficacy of prebiotics, probiotics, synbiotics and antibiotics in irritable bowel syndrome. *Alimentary Pharmacology & Therapeutics*, **48**(10), 1044-1060. <https://doi.org/10.1111/apt.15001>

**Goldstein, R., Braverman, D., and Stankiewicz, H.** (2000). Carbohydrate malabsorption and the effect of dietary restriction on symptoms of irritable bowel syndrome and functional bowel complaints. *Israel Medical Association Journal*, **2**(8), 583-587.

**Guglielmetti, S., Mora, D., Gschwender, M., and Popp, K.** (2011). Randomised clinical trial: *Bifidobacterium bifidum* MIMBb75 significantly alleviates irritable bowel syndrome and improves quality of life--a double-blind, placebo-controlled study. *Aliment Pharmacol Ther*, **33**(10), 1123-1132. <https://doi.org/10.1111/j.1365-2036.2011.04633.x>

**Hajiani, E., Masjedizadeh, A., Shayesteh, A. A., Babazadeh, S., and Seyedian, S. S.** (2019). Comparison between gluten-free regime and regime with gluten in symptoms of patients with irritable bowel syndrome (IBS). *Journal of family medicine and primary care*, **8**(5), 1691-1695. <https://doi.org/10.4103/jfmpc.jfmpc_464_18>

**Halmos, E. P., Power, V. A., Shepherd, S. J., Gibson, P. R., and Muir, J. G.** (2014). A diet low in FODMAPs reduces symptoms of irritable bowel syndrome. *Gastroenterology*, **146**(1), 67-75.e65. <https://doi.org/10.1053/j.gastro.2013.09.046>

**Harvie, R. M., Chisholm, A. W., Bisanz, J. E., Burton, J. P., Herbison, P., Schultz, K., and Schultz, M.** (2017). Long-term irritable bowel syndrome symptom control with reintroduction of selected FODMAPs. *World Journal of Gastroenterology*, **23**(25), 4632-4643. <https://doi.org/10.3748/wjg.v23.i25.4632>

**Holvoet, T., Joossens, M., Vázquez-Castellanos, J. F., Christiaens, E., Heyerick, L., Boelens, J., Verhasselt, B., van Vlierberghe, H., De Vos, M., Raes, J., and De Looze, D.** (2021). Fecal microbiota transplantation reduces symptoms in some patients with irritable bowel syndrome with predominant abdominal bloating: short- and long-term results rrom a placebo-controlled randomized trial. *Gastroenterology*, **160**(1), 145-157.e148. <https://doi.org/10.1053/j.gastro.2020.07.013>

**Johnsen, P. H., Hilpüsch, F., Cavanagh, J. P., Leikanger, I. S., Kolstad, C., Valle, P. C., and Goll, R.** (2018). Faecal microbiota transplantation versus placebo for moderate-to-severe irritable bowel syndrome: a double-blind, randomised, placebo-controlled, parallel-group, single-centre trial. *The Lancet Gastroenterology & Hepatology*, **3**(1), 17-24. <https://doi.org/10.1016/S2468-1253(17)30338-2>

**Kajander, K., Hatakka, K., Poussa, T., Färkkilä, M., and Korpela, R.** (2005). A probiotic mixture alleviates symptoms in irritable bowel syndrome patients: a controlled 6-month intervention. *Alimentary Pharmacology & Therapeutics*, **22**(5), 387-394. <https://doi.org/https://doi.org/10.1111/j.1365-2036.2005.02579.x>

**Kim, J. Y., Park, Y. J., Lee, H. J., Park, M. Y., and Kwon, O.** (2018). Effect of Lactobacillus gasseri BNR17 on irritable bowel syndrome: a randomized, double-blind, placebo-controlled, dose-finding trial. *Food Sci Biotechnol*, **27**(3), 853-857. <https://doi.org/10.1007/s10068-017-0296-7>

**Lembo, A., Pimentel, M., Rao, S. S., Schoenfeld, P., Cash, B., Weinstock, L. B., Paterson, C., Bortey, E., and Forbes, W. P.** (2016). Repeat treatment with rifaximin is safe and effective in patients with diarrhea-predominant irritable bowel syndrome. *Gastroenterology*, **151**(6), 1113-1121. <https://doi.org/10.1053/j.gastro.2016.08.003>

**Lembo, A., Rao, S. S. C., Heimanson, Z., and Pimentel, M.** (2020). Abdominal pain response to rifaximin in patients with irritable bowel syndrome with diarrhea. *Clinical and Translational Gastroenterology*, **11**(3), e00144. <https://doi.org/10.14309/ctg.0000000000000144>

**Ligaarden, S. C., Axelsson, L., Naterstad, K., Lydersen, S., and Farup, P. G.** (2010). A candidate probiotic with unfavourable effects in subjects with irritable bowel syndrome: a randomised controlled trial. *BMC Gastroenterol*, **10**, 16. <https://doi.org/10.1186/1471-230x-10-16>

**Maagaard, L., Ankersen, D. V., Végh, Z., Burisch, J., Jensen, L., Pedersen, N., and Munkholm, P.** (2016). Follow-up of patients with functional bowel symptoms treated with a low FODMAP diet. *World Journal of Gastroenterology*, **22**(15), 4009-4019. <https://doi.org/10.3748/wjg.v22.i15.4009>

**Niv, E., Halak, A., Tiommny, E., Yanai, H., Strul, H., Naftali, T., and Vaisman, N.** (2016). Randomized clinical study: Partially hydrolyzed guar gum (PHGG) versus placebo in the treatment of patients with irritable bowel syndrome. *Nutrition & Metabolism*, **13**(1), 10. <https://doi.org/10.1186/s12986-016-0070-5>

**O'Keeffe, M., Jansen, C., Martin, L., Williams, M., Seamark, L., Staudacher, H. M., Irving, P. M., Whelan, K., and Lomer, M. C.** (2018). Long-term impact of the low-FODMAP diet on gastrointestinal symptoms, dietary intake, patient acceptability, and healthcare utilization in irritable bowel syndrome. *Neurogastroenterology & Motility*, **30**(1), e13154. <https://doi.org/10.1111/nmo.13154>

**Ong, D. K., Mitchell, S. B., Barrett, J. S., Shepherd, S. J., Irving, P. M., Biesiekierski, J. R., Smith, S., Gibson, P. R., and Muir, J. G.** (2010). Manipulation of dietary short chain carbohydrates alters the pattern of gas production and genesis of symptoms in irritable bowel syndrome. *Journal of Gastroenterology and Hepatology*, **25**(8), 1366-1373. <https://doi.org/https://doi.org/10.1111/j.1440-1746.2010.06370.x>

**Paduano, D., Cingolani, A., Tanda, E., and Usai, P.** (2019). Effect of Three Diets (Low-FODMAP, Gluten-free and Balanced) on Irritable Bowel Syndrome Symptoms and Health-Related Quality of Life. *Nutrients*, **11**(7), 1566. <https://www.mdpi.com/2072-6643/11/7/1566>

**Patcharatrakul, T., Juntrapirat, A., Lakananurak, N., and Gonlachanvit, S.** (2019). Effect of Structural Individual Low-FODMAP Dietary Advice vs. Brief Advice on a Commonly Recommended Diet on IBS Symptoms and Intestinal Gas Production. *Nutrients*, **11**(12). <https://doi.org/10.3390/nu11122856>

**Pedersen, N., Andersen, N. N., Végh, Z., Jensen, L., Ankersen, D. V., Felding, M., Simonsen, M. H., Burisch, J., and Munkholm, P.** (2014). Ehealth: low FODMAP diet vs Lactobacillus rhamnosus GG in irritable bowel syndrome. *World Journal of Gastroenterology*, **20**(43), 16215-16226. <https://doi.org/10.3748/wjg.v20.i43.16215>

**Pedersen, N., Vegh, Z., Burisch, J., Jensen, L., Ankersen, D. V., Felding, M., Andersen, N. N., and Munkholm, P.** (2014). Ehealth monitoring in irritable bowel syndrome patients treated with low fermentable oligo-, di-, mono-saccharides and polyols diet. *World Journal of Gastroenterology*, **20**(21), 6680-6684. <https://doi.org/10.3748/wjg.v20.i21.6680>

**Peters, S. L., Biesiekierski, J. R., Yelland, G. W., Muir, J. G., and Gibson, P. R.** (2014). Randomised clinical trial: gluten may cause depression in subjects with non-coeliac gluten sensitivity – an exploratory clinical study. *Alimentary Pharmacology & Therapeutics*, **39**(10), 1104-1112. <https://doi.org/https://doi.org/10.1111/apt.12730>

**Pimentel, M., Lembo, A., Chey, W. D., Zakko, S., Ringel, Y., Yu, J., Mareya, S. M., Shaw, A. L., Bortey, E., and Forbes, W. P.** (2011). Rifaximin Therapy for Patients with Irritable Bowel Syndrome without Constipation. *New England Journal of Medicine*, **364**(1), 22-32. <https://doi.org/10.1056/NEJMoa1004409>

**Pimentel, M., Park, S., Mirocha, J., Kane, S. V., and Kong, Y.** (2006). The effect of a nonabsorbed oral antibiotic (rifaximin) on the symptoms of the irritable bowel syndrome: a randomized trial. *Annals of Internal Medicine*, **145**(8), 557-563. <https://doi.org/10.7326/0003-4819-145-8-200610170-00004>

**Pinto-Sanchez, M. I., Nardelli, A., Borojevic, R., De Palma, G., Calo, N. C., McCarville, J., Caminero, A., Basra, D., Mordhorst, A., Ignatova, E., Hansen, S., Uhde, M., Norman, G. L., Murray, J. A., Smecuol, E., Armstrong, D., Bai, J. C., Schuppan, D., Collins, S. M., Alaedini, A., Moayyedi, P., Verdu, E. F., and Bercik, P.** (2020). Gluten-free diet reduces symptoms, particularly diarrhea, in patients with irritable bowel syndrome and antigliadin IgG. *Clinical Gastroenterology and Hepatology*. <https://doi.org/10.1016/j.cgh.2020.08.040>

**Pirkola, L., Laatikainen, R., Loponen, J., Hongisto, S. M., Hillilä, M., Nuora, A., Yang, B., Linderborg, K. M., and Freese, R.** (2018). Low-FODMAP vs regular rye bread in irritable bowel syndrome: randomized SmartPill(®) study. *World Journal of Gastroenterology*, **24**(11), 1259-1268. <https://doi.org/10.3748/wjg.v24.i11.1259>

**Ringel-Kulka, T., Palsson, O. S., Maier, D., Carroll, I., Galanko, J. A., Leyer, G., and Ringel, Y.** (2011). Probiotic bacteria Lactobacillus acidophilus NCFM and Bifidobacterium lactis Bi-07 versus placebo for the symptoms of bloating in patients with functional bowel disorders: a double-blind study. *Journal of Clinical Gastroenterology*, **45**(6), 518-525. <https://doi.org/10.1097/MCG.0b013e31820ca4d6>

**Saidi, K., Nilholm, C., Roth, B., and Ohlsson, B.** (2021). A carbohydrate-restricted diet for patients with irritable bowel syndrome lowers serum C-peptide, insulin, and leptin without any correlation with symptom reduction. *Nutrition Research*, **86**, 23-36. <https://doi.org/10.1016/j.nutres.2020.12.001>

**Schumann, D., Langhorst, J., Dobos, G., and Cramer, H.** (2018). Randomised clinical trial: yoga vs a low-FODMAP diet in patients with irritable bowel syndrome. *Alimentary Pharmacology & Therapeutics*, **47**(2), 203-211. <https://doi.org/10.1111/apt.14400>

**Shahbazkhani, B., Sadeghi, A., Malekzadeh, R., Khatavi, F., Etemadi, M., Kalantri, E., Rostami-Nejad, M., and Rostami, K.** (2015). Non-celiac gluten sensitivity has narrowed the spectrum of irritable bowel syndrome: a double-blind randomized placebo-controlled trial. *Nutrients*, **7**(6), 4542-4554. <https://doi.org/10.3390/nu7064542>

**Staudacher, H. M., Lomer, M. C., Anderson, J. L., Barrett, J. S., Muir, J. G., Irving, P. M., and Whelan, K.** (2012). Fermentable carbohydrate restriction reduces luminal bifidobacteria and gastrointestinal symptoms in patients with irritable bowel syndrome. *Journal of Nutrition*, **142**(8), 1510-1518. <https://doi.org/10.3945/jn.112.159285>

**Staudacher, H. M., Whelan, K., Irving, P. M., and Lomer, M. C. E.** (2011). Comparison of symptom response following advice for a diet low in fermentable carbohydrates (FODMAPs) versus standard dietary advice in patients with irritable bowel syndrome. *Journal of Human Nutrition and Dietetics*, **24**(5), 487-495. <https://doi.org/https://doi.org/10.1111/j.1365-277X.2011.01162.x>

**van Lanen, A. S., de Bree, A., and Greyling, A.** (2021). Efficacy of a low-FODMAP diet in adult irritable bowel syndrome: a systematic review and meta-analysis. *European Journal of Nutrition*, **60**(6), 3505-3522. <https://doi.org/10.1007/s00394-020-02473-0>

**Vazquez-Roque, M. I., Camilleri, M., Smyrk, T., Murray, J. A., Marietta, E., O'Neill, J., Carlson, P., Lamsam, J., Janzow, D., Eckert, D., Burton, D., and Zinsmeister, A. R.** (2013). A controlled trial of gluten-free diet in patients with irritable bowel syndrome-diarrhea: effects on bowel frequency and intestinal function. *Gastroenterology*, **144**(5), 903-911.e903. <https://doi.org/10.1053/j.gastro.2013.01.049>

**Vernia, P., Ricciardi, M. R., Frandina, C., Bilotta, T., and Frieri, G.** (1995). Lactose malabsorption and irritable bowel syndrome. Effect of a long-term lactose-free diet. *The Italian journal of gastroenterology*, **27**(3), 117-121.

**Weynants, A., Goossens, L., Genetello, M., De Looze, D., and Van Winckel, M.** (2020). The long-term effect and adherence of a low fermentable oligosaccharides disaccharides monosaccharides and polyols (FODMAP) diet in patients with irritable bowel syndrome. *Journal of Human Nutrition and Dietetics*, **33**(2), 159-169. <https://doi.org/https://doi.org/10.1111/jhn.12706>

**Zahedi, M. J., Behrouz, V., and Azimi, M.** (2018). Low fermentable oligo-di-mono-saccharides and polyols diet versus general dietary advice in patients with diarrhea-predominant irritable bowel syndrome: a randomized controlled trial. *Journal of Gastroenterology and Hepatology*, **33**(6), 1192-1199. <https://doi.org/https://doi.org/10.1111/jgh.14051>

**Zanwar, V. G., Pawar, S. V., Gambhire, P. A., Jain, S. S., Surude, R. G., Shah, V. B., Contractor, Q. Q., and Rathi, P. M.** (2016). Symptomatic improvement with gluten restriction in irritable bowel syndrome: a prospective, randomized, double blinded placebo controlled trial. *Intestinal Research*, **14**(4), 343-350. <https://doi.org/10.5217/ir.2016.14.4.343>

**Zhang, Y., Feng, L., Wang, X., Fox, M., Luo, L., Du, L., Chen, B., Chen, X., He, H., Zhu, S., Hu, Z., Chen, S., Long, Y., Zhu, Y., Xu, L., Deng, Y., Misselwitz, B., Lang, B. M., Yilmaz, B., Kim, J. J., Owyang, C., and Dai, N.** (2021). Low fermentable oligosaccharides, disaccharides, monosaccharides, and polyols diet compared with traditional dietary advice for diarrhea-predominant irritable bowel syndrome: a parallel-group, randomized controlled trial with analysis of clinical and microbiological factors associated with patient outcomes. *The American Journal of Clinical Nutrition*, **113**(6), 1531-1545. <https://doi.org/10.1093/ajcn/nqab005>
